# Party affiliation, political ideology, views on democracy and society, and support for political violence in the United States of America: findings from a nationally representative survey

**DOI:** 10.1371/journal.pone.0355678

**Published:** 2026-09-16

**Authors:** Garen J. Wintemute, Andrew Crawford, Sonia L. Robinson, Daniel Tancredi, Elizabeth A. Tomsich, Julia P. Schleimer, Paul M. Reeping, Aaron B. Shev, Bradley Velasquez, Veronica A. Pear

**Affiliations:** 1 Department of Emergency Medicine, University of California Davis School of Medicine, Sacramento, California, United States of America; 2 Department of Pediatrics, University of California Davis School of Medicine, Sacramento, California, United States of America; Public Library of Science, UNITED KINGDOM OF GREAT BRITAIN AND NORTHERN IRELAND

## Abstract

**Background:**

Recent research in the United States has found concerning levels of support for and willingness to engage in political violence and for extreme beliefs linked to violence. Associations between political party affiliation and political ideology and these levels of support have not been quantified.

**Methods:**

Cross-sectional nationally representative survey conducted May 13 to June 2, 2022, of adult members of the Ipsos KnowledgePanel. Primary outcomes concerned support for and personal willingness to engage in political violence, presented as prevalences (weighted to achieve representativeness) with 95% confidence intervals (CI) and prevalence differences (adjusted for sociodemographics and geography) with p-values adjusted for the false discovery rate and reported as q-values.

**Results:**

The completion rate was 55.8%; the analytic sample included 8,620 respondents. Higher percentages of Republicans (from 35.3% to 49.5%) than Democrats (from 24.7% to 26.7%) considered violence to be usually or always justified to advance at least 1 of 17 specific political objectives. Few respondents (<3%), with no statistically significant differences by party affiliation, were “very or completely willing” to threaten or intimidate, injure, or kill another person for political purposes. Strong Republicans were more likely than strong Democrats to consider it “very or extremely likely” that they would be armed in a future situation where they considered political violence to be justified: 14.6% (95% CI, 12.5%, 17.0%) and 4.7% (95% CI, 3.4%, 6.3%), respectively; adjusted prevalence difference 9.7% (95% CI, 6.9%, 12.1%; q < 0.001). Respondents rarely (<2%), and again without differences by party affiliation, thought it very or extremely likely that they would shoot someone in such a situation. Findings for political ideology resembled those for party affiliation.

**Conclusions:**

Republicans and conservatives were more likely than Democrats and liberals to view political violence as justified to advance specific political objectives; this finding is a function of the specific objectives that were presented. Republicans were not more willing than Democrats to engage in political violence themselves. These findings deepen our understanding of the potential threat posed by political violence in the United States and can contribute to prevention efforts.

## Introduction

Findings from recent public opinion polls [1–4] and survey research [5–8], statements by elected officials [9,10], assessments by experts in several disciplines [11–13], and the report of the House Select Committee to Investigate the January 6th Attack on the United States Capitol [14] have raised concern about the stability of democracy and the prospect of widespread political violence—the use of physical force to advance political objectives [15]—in the United States (US).

“Political violence,” as used here, is a broad term that can include targeted violence (directed at a group defined by characteristics such as perceived race and ethnicity, sexual orientation, religion, or national origin) and terrorist violence (which may be directed against the population in general). Like other forms of violence, political violence can properly be described as a public health problem [16]. Political violence kills and injures people and has many other negative population-level effects [17,18]; its incidence has increased in recent years in the US [11]. Given a high prevalence of firearm ownership in the US and increased support for political violence among subsets of firearm owners [19], there is specific concern for the possibility of firearm-involved political violence.

Adopting a public health approach [20], which relies on systematic investigative techniques that were originally developed to address infectious and chronic diseases and intervention guidelines that were developed to address other forms of injury [21,22], will likely prove beneficial for understanding and preventing political violence—as it already has for firearm violence [23] and motor vehicle injuries [24,25].

Working from that public health perspective, in late spring 2022 we conducted the Life in America Survey to develop a better understanding of political violence based on data from a large nationally representative sample. The first report from the survey presented the broad contours of the problem [26]. We found concerningly high levels of support for extreme beliefs and the use of violence to advance political objectives in the US—including personal willingness to engage in that violence. Both support for and willingness to engage in political violence were more common among men than women and were inversely related to respondents’ education and income.

In this study we seek to characterize variation in support for those extreme beliefs and for political violence in association with 2 related exposures of interest: the broad ranges of self-reported political party affiliation (from strong Democrat to strong Republican) and political ideology (from extremely liberal to extremely conservative). This study builds upon and extends our findings from the survey on support for political violence among so-called MAGA Republicans, in which the comparison of greatest interest was to other members of that same political party [27].

Most modern studies of party affiliation and support for political violence have found higher support for violence among Republicans than Democrats [1,3–7,28–30], with support particularly high among those who identify as strong Republicans [6], but there have been exceptions [31–33]. One study suggested that increased support for political violence among Republicans relative to Democrats may have developed only in the past few years [7].

These studies have largely involved small samples or addressed violence only against members of an opposing political party. They have generally not defined violence, asked about support for non-political violence (to assess the specificity of support for political violence) or violence to advance specific political objectives, or explored personal willingness to engage in political violence [8]. This study advances our understanding by proceeding from a specific definition of violence, broadening the scope of the inquiry beyond partisan violence to include many forms of political violence, establishing a context for support of political violence by assessing support for other forms of violence as well, probing specifically on the possibility of the use of firearms in political violence, and—perhaps most importantly—by assessing respondents’ self-reported willingness to engage in political violence themselves. The findings are likely to have direct implications for prevention efforts.

## Methods

The 2022 Life in America Survey was designed by the authors and administered online in English and Spanish from May 13 to June 2, 2022 by the survey research firm Ipsos [34]. Written or verbal consent was not required for participation in this online survey; before participants accessed the questionnaire, they were provided informed consent information that concluded, “[by] continuing, you are agreeing to participate in this study.” The study was approved by the University of California Davis Institutional Review Board (protocol 187125: exempt from full review, category 2, survey research) without a consent requirement and is reported following American Association for Public Opinion Research guidelines [35].

### Participants

Respondents were drawn from the Ipsos KnowledgePanel, an online research panel that has been widely used in population-based research, including studies of violence and firearm ownership [36–41]. Studies by Ipsos and others have found that KnowledgePanel surveys yield findings similar to those from random digit dial samples, with little evidence of recruitment bias (difference between individuals who agree to or decline recruitment into KnowledgePanel), or self-selection bias (difference between KnowledgePanel members who respond or do not respond to an invitation to participate in a specific survey, including difference on stigmatized behaviors that are the topic of the survey) [42].

To establish a nationally representative panel, members are recruited on an ongoing basis through address-based probability sampling using data from the US Postal Service’s Delivery Sequence File [42,43]. Recruited adults in households without internet access are provided a web-enabled device and free internet service, and a modest, primarily points-based incentive program seeks to encourage participation and promote participants’ retention in KnowledgePanel over time.

A probability-proportional-to-size procedure was used to select a study-specific sample for this survey. All panel members who were aged 18 years and older were eligible for selection. Invitations were sent by e-mail; automatic reminders were delivered to non-respondents by e-mail and telephone beginning 3 days later [43].

A final survey weight variable provided by Ipsos adjusted for the initial probability of selection into KnowledgePanel and for survey-specific nonresponse and over- or under-coverage using design weights with post-stratification raking ratio adjustments. With weighting, the sample is designed to be representative of the noninstitutionalized adult population of the US as reflected in the 2021 March supplement of the Current Population Survey [42,44,45].

### Measures

#### Party affiliation and political ideology.

Sociodemographic data, including political party affiliation and political ideology, were collected by Ipsos from profiles created and maintained by KnowledgePanel members. Party affiliation was derived following a procedure used since the 1950s by the American National Election Studies [46]. As described in the report of a previous KnowledgePanel survey,

Respondents were first asked, “Generally speaking, do you think of yourself as” (response choices: “Republican,” “Democrat,” “Independent,” “Another party,” “No preference”). Republicans/Democrats were then asked, “Would you call yourself a” (response choices: “Strong Republican/Democrat,” “Not very strong Republican/Democrat”). All others were asked the follow-up question: “Do you think of yourself as closer to the” (response choices: “Republican Party,” “Democratic Party”) [47].

Respondents who answered that follow-up question were coded by Ipsos as “Leans Republican/Democrat.” Respondents who refused to answer (n = 309, 3.6% of the sample) were coded by Ipsos as “undecided/independent/other.”

Political ideology was reported by panel members in responding to the question, “In general, do you think of yourself as…” with 7 response options ranging from “extremely liberal” to “extremely conservative” and with “moderate/middle of the road” as a midpoint.

#### Beliefs.

Survey questions in this area addressed beliefs regarding democracy, such as whether it is the preferred form of government for the US; the potential need for violence in the US; and beliefs about race and ethnicity, religion, and American society. Selected questions from prior surveys were included or adapted to track trends in opinion and provide context for responses to questions that had not been asked previously.

Support for the QAnon delusion complex was determined through respondents’ agreement with statements of core elements of that complex: that US institutions “are controlled by a group of Satan-worshipping pedophiles who run a global child sex trafficking operation” and that “a storm coming soon” will “sweep away the elites in power and restore the rightful leaders.” Support for the great-replacement belief [48] was determined through respondents’ agreement with the statement that “in America, native-born white people are being replaced by immigrants.”

#### Violence.

Violence was represented by the phrase “force or violence,” defined as “physical force strong enough that it could cause pain or injury to a person.” “Force or violence to advance an important political objective that you support” was used in questions about respondents’ support for and willingness to engage in political violence.

Questions on violence began with its use in non-political situations; these questions were included to assess whether differences seen in support for political violence were specific to political violence or were a manifestation of more generalized support for violence as a means of resolving interpersonal social problems and disputes. The items were presented in a fixed order that, in the judgment of the authors, proceeded from more likely to less likely to be seen by respondents as justifying violence: from “in self-defense” to “to get respect.” This was done to create an expected response transition from support to nonsupport for violence that respondents would need to reverse to indicate support for political violence.

Respondents were then asked about the extent to which they considered political violence to be justified “in general” and to advance specified political objectives; examples include “to return Donald Trump to the presidency this year,” “to preserve an American way of life based on Western European traditions,” and “to stop police violence.” There were 17 objectives; our intent in selecting them was to obtain responses on a wide array of specific possibilities. Some measures related to electoral violence; others addressed targeted violence (defined by the intended victims) or violence to accomplish narrow objectives; and still others concerned violence to bring about (or prevent) social change more broadly. Some were chosen to be of interest primarily to participants on one side or the other of the partisan and ideological spectra, and others were chosen for a lack of expected association with party affiliation and ideology. Nine objectives were presented to all respondents and 8 were paired, with each respondent seeing only 1 item from each pair; each respondent was presented with 13 of 17 objectives.

Respondents who considered political violence to be at least sometimes justified for at least 1 of these objectives were asked about their personal willingness to engage in political violence: by type of violence (to “damage property,” “threaten or intimidate a person,” “injure a person,” “kill a person”) and against members of 9 target populations (examples: “an elected federal or state government official,” “a police officer,” “a person who does not share your religion”).

Finally, all respondents were asked about the likelihood of their future use of firearms in connection with political violence in a situation where they considered such violence justified (e.g., “I will be armed with a gun,” “I will shoot someone with a gun”).

The full text of all questions reported on in this study is provided in the supplement (see Supplementary information).

### Implementation

Ipsos translated the questionnaire into Spanish, and interpreting services staff at UC Davis Medical Center reviewed the translation. Forty KnowledgePanel members participated in an English language pretest administered April 27 to May 2, 2022.

Respondents were randomized 1:1 to receive response options in order from negative to positive valence or the reverse throughout the questionnaire. Where a question presented multiple statements for respondents to consider, the order in which those statements were presented was randomized unless ordering was necessary. To reduce response bias, logic-driving questions (those to which responses might invoke a skip pattern) included non-response prompts. The questionnaire included a check for inattentiveness among respondents: two fictitious entries were included in lists of news and information outlets for which respondents provided information on their extent of use.

We employed unipolar response arrays without a neutral midpoint (e.g., do not agree, somewhat agree, strongly agree, very strongly agree). The literature is not in agreement on whether such midpoints should be included [8,49]. We were persuaded by the studies reviewed by Chyung et al. [49], which suggest that such midpoints facilitate selection of “a minimally acceptable response as soon as it is found,” known as satisficing. Our analyses focus on responses above the “somewhat” or “sometimes” level to minimize the impact of potential satisficing on the results. According to Chyung et al. [49], satisficing is particularly common when respondents are uncomfortable with the topics of the survey or under social desirability pressures; both conditions apply here.

### Statistical analysis

To generate prevalence estimates, we calculated percentages weighted to achieve national representativeness and their 95% confidence intervals (CI) using PROC SURVEYFREQ in SAS version 9.4 (SAS Institute, Inc., Cary, NC) and Complex Samples Frequencies in IBM SPSS Statistics, version 28 (IBM Corp., Armonk, NY).

To compute unadjusted and adjusted prevalence differences (aPDs) and their 95% CIs, we defined outcomes dichotomously (such as by combining “very willing” with “completely willing” responses and “not willing” with “somewhat willing” responses in 2 categories) and used PROC SURVEYREG, employing robust standard errors to correct for design effects and heteroskedasticity in binary outcomes. We considered several models, choosing the final model based on concordance with theory, findings from prior research, and fit statistics. This model adjusted for age, race and ethnicity, gender, education, income, Census division, and rurality. We also considered an unadjusted model; a model that adjusted for age, race and ethnicity, and gender; and a model adjusting for those variables and income, education, and Census division.

P-values were corrected for multiple comparisons by controlling the false discovery rate (FDR) using the Benjamini-Hochberg method [50]. The corrected values are known as FDR-adjusted (or FDR-corrected) p-values or as q-values [51]; we employ the latter term here. Q-values represent the probability that the given difference would be a false discovery; they represent the expected proportion of “false positives” that would be seen among the collection of all differences whose q-values were at or below the given q-value.

For comparisons across the entire spectrum of party affiliation and political ideology, we chose midpoints as referents in regression models; these findings are presented in the tables. For political ideology, the midpoint was the “moderate/middle of the road” stratum. For party affiliation, the midpoint was the combination of the small “undecided/independent/other” stratum with the “leans Republican” and “leans Democrat” strata. We made this combination after exploratory analysis showed substantial overlap between this aggregate and the “moderate/middle of the road” political ideology stratum (S1 Table in S1 File).

We also directly compared strong Republicans with strong Democrats on some outcomes, again using adjusted prevalence differences and q-values. These comparisons are presented in the text with a visual summary included for key political violence measures.

In a sensitivity analysis, we excluded the 447 inattentive respondents who reported use of one or both fictitious news sources. We compared responses from attentives and inattentives on other items associated with risk for violence (such as a prior history of arrest for violent crimes) using weighted percentages as described above and assessed the significance of differences with likelihood-ratio tests.

### Ethics approval

This study was approved by the University of California Davis Institutional Review Board. The University of California, Davis, in accordance with its FWA with the Department of Health & Human Services, adheres to all federal and state regulations related to the protection of human research subjects, including 45 CFR 46 (“The Common Rule”), 21 CFR 50, 21 CFR 56 for FDA regulated products, and the principles of The Belmont Report and Institutional policies and procedures. In addition, the International Conference on Harmonization, Good Clinical Practice (ICH GCP) principles are adhered to insofar as they parallel the previously mentioned regulations and policies.

## Results

Of 15,449 panel members invited to participate, 8,620 completed the survey, yielding a 55.8% completion rate, and another 730 (4.7%) members opened the survey but did not proceed. After weighting, half of the respondents (50.5%, 95% CI 49.3%, 51.7%) were female; 62.6% (95% CI 61.4%, 63.9%) were white, non-Hispanic; mean (SD) age was 48.4 (18.0) years. Demographic descriptions of respondents by party affiliation and political ideology are in S2 and S3 Tables in S1 File; descriptions of respondents and nonrespondents are in S4 Table in S1 File.

### Findings for party affiliation

#### Democracy, the potential need for violence, and civil war.

Majorities of respondents across the spectrum of party affiliation (ranging from 60.4% to 82.8% for Republicans and from 54.7% to 79.3% for Democrats) perceived “a serious threat to our democracy” and believed it very or extremely important “for the United States to remain a democracy” (from 92.7% to 93.5% for Republicans and from 89.8% to 95.1% for Democrats) (S5 Table in S1 File). While majorities agreed strongly or very strongly that “democracy is the best form of government” (from 75.2% to 77.8% for Republicans and from 66.8% to 79.1% for Democrats), large minorities of respondents agreed strongly or very strongly that “American democracy only serves the interests of the wealthy and powerful” (from 28.1% to 29.1% for Republicans and from 35.7% to 40.8% for Democrats).

Strong Republicans were more likely than strong Democrats to agree strongly or very strongly that “having a strong leader for America is more important than having a democracy” (S5 Table in S1 File) (aPD, 11.3%, 95% CI 7.9%, 14.7%; q < 0.001). Strong Republicans were also more likely than strong Democrats to agree strongly or very strongly that “armed citizens should patrol polling places at election time” (aPD 10.7%, 95% CI 8.3%, 13.2%; q < 0.001).

Strong Republicans were more likely than strong Democrats (Table 1) to agree strongly or very strongly that force or violence might be justified under 3 sets of circumstances: 1) to “protect American democracy…if elected leaders will not” (aPD 16.0%, 95% CI 12.6%, 19.4%; q < 0.001), 2) to save “our American way of life” that is “disappearing so fast” (aPD 21.4%, 95% CI 18.1%, 24.7%; q < 0.001), and 3) to “save our country” because “things have gotten so far off track” (aPD 13.7%, 95% CI 11.1%, 16.4%; q < 0.001). Strong Republicans were also more likely than strong Democrats to agree strongly or very strongly that “in the next few years, there will be civil war in the United States” (aPD 7.8%, 95% CI 4.7%, 11.0%; q < 0.001).

**Table 1:** Party affiliation and beliefs concerning the potential need for violence in the US.

Statement	Party Affiliation									
	Strong Democrat		Not Very Strong Democrat		Undecided/Independent/Other/Leans		Not Very Strong Republican		Strong Republican	
	Unweighted n	Weighted % (95% CI)	Unweighted n	Weighted % (95% CI)	Unweighted n	Weighted % (95% CI)	Unweighted n	Weighted % (95% CI)	Unweighted n	Weighted % (95% CI)
If elected leaders will not protect American democracy, the people must do it themselves, even if it requires taking violent actions.										
Do not agree	1050	61.9 (59.1, 64.6)	694	60.7 (57.3, 63.9)	1791	50.1 (48.2, 52.0)	447	45.3 (41.8, 48.9)	512	35.6 (32.9, 38.4)
Somewhat agree	372	25.0 (22.7, 27.6)	263	25.7 (22.8, 28.7)	1024	30.4 (28.6, 32.1)	322	37.5 (34.0, 41.1)	483	34.2 (31.5, 37.0)
Strongly or very strongly agree	174	13.1 (11.2, 15.3)	130	13.7 (11.5, 16.3)	641	19.6 (18.1, 21.1)	149	17.2 (14.6, 20.2)	421	30.2 (27.6, 33.0)
Adjusted prevalence difference (95% CI; q-value)	−3.9 (−6.4, −1.3; 0.01)		−4.4 (−7.3, −1.6; 0.007)		Referent		−0.8 (−3.9, 2.4; 0.73)		12.1 (9.0, 15.3; < 0.001)	
Our American way of life is disappearing so fast that we may have to use force to save it.										
Do not agree	1250	75.3 (72.7, 77.7)	738	66.5 (63.2, 69.6)	2073	57.7 (55.8, 59.6)	445	46.3 (42.8, 49.9)	443	29.7 (27.2, 32.3)
Somewhat agree	231	15.0 (13.1, 17.1)	254	23.9 (21.2, 26.9)	875	26.9 (25.2, 28.6)	325	37.3 (33.9, 40.9)	527	37.7 (35.0, 40.5)
Strongly or very strongly agree	121	9.7 (7.9, 11.8)	93	9.6 (7.7, 11.9)	506	15.4 (14.1, 16.9)	146	16.4 (13.9, 19.3)	446	32.6 (29.9, 35.4)
Adjusted prevalence difference (95% CI; q-value)	−3.9 (−6.2, −1.7; 0.003)		−5.1 (−7.6, −2.6; < 0.001)		Referent		2.0 (−1.0, 5.0; 0.29)		17.5 (14.1, 20.6; < 0.001)	
Because things have gotten so far off track, true American patriots may have to resort to violence in order to save our country.										
Do not agree	1446	87.6 (85.3, 89.5)	909	82.1 (79.3, 84.6)	2605	73.8 (72.0, 75.5)	667	70.6 (67.1, 73.9)	759	52.6 (49.7, 55.4)
Somewhat agree	108	8.5 (6.8, 10.4)	128	12.5 (10.5, 15.0)	583	18.3 (16.8, 19.9)	176	21.8 (18.8, 25.1)	426	29.9 (27.4, 32.6)
Strongly agree	44	4.0 (2.9, 5.5)	49	5.3 (4.0, 7.2)	248	7.9 (6.9, 9.0)	70	7.6 (6.0, 9.7)	236	17.5 (15.4, 19.9)
Adjusted prevalence difference (95% CI; q-value)	−2.7 (−4.3, −1.0; 0.005)		−2.2 (−4.2, −0.3; 0.06)		Referent		1.1 (−1.1, 3.2; 0.44)		11.0 (8.5, 13.6; < 0.001)	
In the next few years, there will be civil war in the United States.										
Do not agree	883	53.3 (50.5, 56.1)	590	53.3 (49.9, 56.6)	1753	49.1 (47.2, 51.0)	474	49.2 (45.6, 52.8)	557	37.8 (35.1, 40.6)
Somewhat agree	558	34.6 (32.0, 37.2)	393	36.2 (33.1, 39.5)	1235	36.7 (34.8, 38.5)	330	37.8 (34.3, 41.4)	605	42.4 (39.6, 45.3)
Strongly or very strongly agree	156	12.1 (10.2, 14.3)	100	10.5 (8.5, 12.8)	446	14.2 (12.9, 15.6)	108	13.0 (10.7, 15.8)	253	19.8 (17.4, 22.3)
Adjusted prevalence difference (95% CI; q-value)	−0.6 (−3.0, 1.8; 0.73)		−3.5 (−6.1, −1.0; 0.02)		Referent		0.4 (−2.4, 3.1; 0.86)		7.2 (4.5, 10.0; < 0.001)	

APDs are for “strongly or very strongly agree” responses and are adjusted for age, race and ethnicity, gender, education, income, Census division, and rurality. Q-values, also known as FDR-adjusted (or FDR-corrected) p-values, represent the probability that the given difference would be a false discovery; they represent the expected proportion of “false positives” that would be seen among the collection of all differences whose q-values were at or below the given q-value.

#### Race and ethnicity and American society.

Five items explored beliefs on race and ethnicity and the great-replacement belief (S6 Table in S1 File). Democrats were far more likely than Republicans to agree strongly or very strongly that “white people benefit from advantages in society that Black people do not have” (ranging from 55.4% to 74.3% for Democrats and from 5.5% to 15.3% for Republicans), that “straight white men hold far too much power in America” (from 39.7% to 62.6% for Democrats and from 6.3% to 13.9% for Republicans), and that “having more Black Americans, Latinos, and Asian Americans is good for the country” (from 55.7% to 71.9% for Democrats and from 19.8% to 29.4% for Republicans).

Conversely, Republicans were far more likely than Democrats to agree strongly or very strongly that “discrimination against whites is as big a problem as discrimination against Blacks and other minorities” (from 37.9% to 60.8% for Republicans and from 8.1% to 13.3% for Democrats) and that “in America, native-born white people are being replaced by immigrants” (from 20.8% to 38.2% for Republicans and from 5.8% to 10.4% for Democrats) (S6 Table in S1 File).

Three items addressed the central elements of the QAnon delusion complex and a related religious belief (S7 Table in S1 File). Strong Republicans were more likely than strong Democrats to agree strongly or very strongly that US institutions “are controlled by a group of Satan-worshipping pedophiles who run a global child sex trafficking operation” (aPD 12.4%, 95% CI 9.6%, 15.2%; q < 0.001) and that “a storm coming soon” will “sweep away the elites in power and restore the rightful leaders” (aPD 13.1%, 95% CI 10.2%, 16.0%; q < 0.001). Strong or very strong agreement with the statement that “the chaos in America today is evidence that we are living in what the Bible calls ‘the end times’” was also more common among strong Republicans than among strong Democrats (aPD 21.0%, 95% CI 17.7%, 24.3%; q < 0.001).

#### Non-political violence.

Respondents’ views on the justifiability of non-political violence varied substantially with circumstance. Most respondents across the range of party affiliation—Republicans more frequently than Democrats—saw violence as usually or always justified in self-defense or to prevent assaultive or self-inflicted injury to others (S8 Table in S1 File). Conversely, at least 80% of respondents across party affiliation reported that violence to win an argument, respond to an insult, or get respect was never justified. Fewer than 6% saw such violence as usually or always justified, and there were no statistically significant differences across party affiliation (S8 Table in S1 File).

#### Political violence.

Support for political violence “in general” was uncommon and did not vary significantly across party affiliation. Fewer than 4% of respondents in any group viewed it as usually or always justified (Table 2).

**Table 2:** Party affiliation and justification for political violence, in general and for 9 specific objectives.

What do you think about the use of force or violence in the following situations?	Party Affiliation									
	Strong Democrat		Not Very Strong Democrat		Undecided/Independent/Other/Leans		Not Very Strong Republican		Strong Republican	
	Unweighted n	Weighted % (95% CI)	Unweighted n	Weighted % (95% CI)	Unweighted n	Weighted % (95% CI)	Unweighted n	Weighted % (95% CI)	Unweighted n	Weighted % (95% CI)
In general...to advance an important political objective that you support										
Never justified	1342	80.5 (78.0, 82.8)	910	80.0 (77.0, 82.6)	2863	78.4 (76.7, 80.0)	761	79.3 (76.0, 82.2)	1177	80.9 (78.4, 83.1)
Sometimes justified	225	15.9 (13.9, 18.2)	153	16.4 (14.0, 19.2)	575	18.9 (17.4, 20.5)	144	17.7 (15.0, 20.8)	228	16.3 (14.2, 18.5)
Usually or always justified	38	3.5 (2.5, 5.0)	31	3.6 (2.5, 5.2)	72	2.7 (2.1, 3.5)	18	3.0 (1.7, 5.1)	29	2.8 (1.8, 4.3)
Adjusted prevalence difference (95% CI; q-value)	1.0 (−0.5, 2.5; 0.29)		0.4 (−1.1, 2.0; 0.69)		Referent		1.2 (−.05, 2.9; 0.27)		1.7 (0.3, 3.1; 0.052)	
										
Usually or always justified to advance at least 1 of 17 objectives	387	26.7 (24.3, 29.4)	265	24.7 (21.9, 27.7)	1075	31.5 (29.8, 33.3)	322	35.3 (31.9, 38.9)	714	49.5 (46.6, 52.3)
Adjusted prevalence difference (95% CI; q-value)	−3.3 (−6.3, −0.2; 0.09)		−6.6 (−10.0, −3.2; < 0.001)		Referent		5.2 (1.4, 9.0; 0.02)		18.4 (15.0, 21.8; < 0.001)	
										
To return Donald Trump to the presidency this year										
Never justified	1509	92.9 (91.0, 94.3)	1010	91.6 (89.4, 93.4)	3141	89.2 (87.9, 90.4)	799	85.4 (82.4, 88.0)	1136	78.8 (76.2, 81.1)
Sometimes justified	38	2.8 (1.9, 4.0)	44	5.3 (3.8, 7.2)	181	6.4 (5.4, 7.5)	64	7.9 (6.1, 10.2)	134	9.4 (7.8, 11.2)
Usually or always justified	48	4.3 (3.2, 5.9)	30	3.1 (2.1, 4.6)	138	4.4 (3.7, 5.3)	50	6.7 (4.9, 9.1)	155	11.9 (10.0, 14.0)
Adjusted prevalence difference (95% CI; q-value)	0.5 (−1.2, 2.1; 0.68)		−1.5 (−2.9, 0.0; 0.11)		Referent		2.9 (0.7, 5.1; 0.02)		8.5 (6.2, 10.7; < 0.001)	
To stop an election from being stolen										
Never justified	1317	81.2 (78.9, 83.4)	885	80.5 (77.7, 83.0)	2662	76.3 (74.6, 77.8)	666	70.6 (67.1, 73.9)	865	60.5 (57.7, 63.3)
Sometimes justified	182	12.0 (10.2, 14.0)	131	12.3 (10.3, 14.6)	564	16.6 (15.2, 18.0)	192	22.5 (19.6, 25.8)	326	22.5 (20.2, 24.9)
Usually or always justified	94	6.8 (5.5, 8.4)	70	7.2 (5.6, 9.2)	240	7.2 (6.3, 8.3)	59	6.8 (5.1, 9.1)	233	17.0 (14.8, 19.4)
Adjusted prevalence difference (95% CI; q-value)	−0.1 (−1.9, 1.7; 0.93)		−0.4 (−2.5, 1.7; 0.80)		Referent		0.4 (−1.7, 2.6; 0.78)		10.2 (7.7, 12.8; < 0.001)	
To stop people who do not share my beliefs from voting										
Never justified	1512	92.6 (90.6, 94.1)	1008	90.8 (88.5, 92.7)	3273	92.8 (91.6, 93.8)	864	92.6 (90.1, 94.5)	1354	94.1 (92.4, 95.4)
Sometimes justified	43	3.7 (2.6, 5.3)	53	6.0 (4.5, 7.9)	139	5.0 (4.2, 6.0)	44	6.1 (4.4, 8.4)	49	3.8 (2.9, 5.1)
Usually or always justified	43	3.7 (2.7, 5.2)	27	3.2 (2.2, 4.8)	61	2.2 (1.7, 2.9)	8	1.3 (0.6, 2.8)	23	2.1 (1.3, 3.4)
Adjusted prevalence difference (95% CI; q-value)	1.6 (0.2, 3.0; 0.055)		0.6 (−0.9, 2.0; 0.55)		Referent		0.4 (−0.8, 1.5; 0.68)		1.7 (0.4, 2.9; 0.02)	
To prevent discrimination based on race or ethnicity										
Never justified	968	58.8 (56.0, 61.5)	713	63.3 (60.0, 66.5)	2275	62.5 (60.7, 64.4)	620	64.8 (61.2, 68.2)	1001	70.1 (67.4, 72.7)
Sometimes justified	469	29.6 (27.2, 32.2)	282	27.3 (24.4, 30.5)	931	28.4 (26.7, 30.2)	246	29.5 (26.2, 33.0)	304	21.3 (19.1, 23.7)
Usually or always justified	157	11.6 (9.8, 13.6)	90	9.4 (7.5, 11.6)	264	9.1 (8.0, 10.3)	51	5.8 (4.2, 7.9)	114	8.6 (7.0, 10.4)
Adjusted prevalence difference (95% CI; q-value)	2.8 (0.6, 5.0; 0.04)		−0.2 (−2.6, 2.2; 0.91)		Referent		−1.6 (−3.7, 0.5; 0.22)		1.8 (−0.3, 3.9; 0.17)	
To preserve an American way of life based on Western European traditions										
Never justified	1370	86.2 (84.1, 88.0)	899	82.7 (80.0, 85.1)	2637	76.5 (74.8, 78.0)	610	67.6 (64.1, 70.9)	822	58.5 (55.7, 61.3)
Sometimes justified	165	10.0 (8.5, 11.8)	146	13.6 (11.4, 16.0)	647	18.2 (16.8, 19.7)	260	27.8 (24.7, 31.2)	440	30.2 (27.6, 32.8)
Usually or always justified	51	3.8 (2.8, 5.2)	37	3.7 (2.6, 5.3)	175	5.3 (4.5, 6.3)	38	4.6 (3.2, 6.5)	151	11.3 (9.5, 13.4)
Adjusted prevalence difference (95% CI; q-value)	−1.0 (−2.5, 0.4; 0.28)		−1.3 (2.9, 0.3; .021)		Referent		0.1 (−1.7, 1.9; 0.94)		6.5 (4.3, 8.7; < 0.001)	
To preserve the American way of life l believe in										
Never justified	1096	68.1 (65.5, 70.6)	695	64.2 (60.9, 67.3)	1986	57.9 (56.0, 59.7)	414	45.4 (41.8, 49.0)	496	35.8 (33.1, 38.7)
Sometimes justified	385	23.9 (21.6, 26.3)	301	26.7 (23.9, 29.7)	1129	31.6 (29.9, 33.4)	386	41.2 (37.7, 44.8)	592	40.3 (37.5, 43.1)
Usually or always justified	117	8.0 (6.6, 9.7)	95	9.1 (7.3, 11.2)	379	10.5 (9.4, 11.7)	119	13.4 (11.1, 16.2)	340	23.8 (21.4, 26.4)
Adjusted prevalence difference (95% CI; q-value)	−1.8 (−3.7, 0.2; 0.14)		−0.9 (−3.2, 1.3; 0.54)		Referent		3.5 (0.8, 6.3; 0.03)		13.2 (10.3, 16.0; < 0.001)	
To oppose Americans who do not share my beliefs										
Never justified	1462	89.2 (87.2, 91.0)	968	86.5 (83.9, 88.7)	3160	88.7 (87.4, 89.9)	838	88.7 (85.9, 91.0)	1314	91.2 (89.3, 92.7)
Sometimes justified	97	7.1 (5.7, 8.9)	86	9.4 (7.6, 11.7)	269	8.8 (7.7, 9.9)	76	9.7 (7.7, 12.1)	91	6.4 (5.1, 7.9)
Usually or always justified	42	3.6 (2.6, 5.1)	36	4.1 (2.9, 5.8)	67	2.6 (1.9, 3.4)	8	1.6 (0.7, 3.7)	26	2.5 (1.6, 3.8)
Adjusted prevalence difference (95% CI; q-value)	1.2 (−0.2, 2.6; 0.18)		1.0 (−0.7, 2.6; 0.36)		Referent		−0.0 (−1.5, 1.4; 0.97)		1.3 (−0.0, 2.6; 0.11)	
To oppose the government when it does not share my beliefs										
Never justified	1388	84.4 (82.1, 86.5)	948	85.0 (82.2, 87.4)	2824	78.9 (77.2, 80.5)	766	82.0 (78.9, 84.8)	1110	77.8 (75.3, 80.1)
Sometimes justified	167	12.3 (10.5, 14.4)	105	11.3 (9.2, 13.8)	537	17.1 (15.7, 18.7)	137	16.3 (13.6, 19.3)	257	18.1 (16.0, 20.4)
Usually or always justified	41	3.3 (2.3, 4.6)	32	3.7 (2.5, 5.4)	111	4.0 (3.2, 4.9)	12	1.7 (0.9, 3.2)	52	4.1 (3.1, 5.6)
Adjusted prevalence difference (95% CI; q-value)	−0.3 (−1.7, 1.1; 0.78)		−0.4 (−2.0, 1.3; 0.76)		Referent		−1.0 (−2.3, 0.3; 0.24)		1.7 (0.2, 3.2; 0.06)	
To oppose the government when it tries to take private land for public purposes										
Never justified	1196	72.8 (70.1, 75.2)	766	69.1 (65.9, 72.2)	2140	60.4 (58.5, 62.3)	533	56.2 (52.6, 59.8)	680	47.4 (44.5, 50.2)
Sometimes justified	313	20.5 (18.3, 22.9)	257	24.9 (22.0, 27.9)	1016	29.4 (27.7, 31.2)	291	32.5 (29.2, 36.0)	543	37.0 (34.3, 39.8)
Usually or always justified	85	6.7 (5.3, 8.4)	58	6.0 (4.6, 7.9)	309	10.2 (9.0, 11.4)	90	11.3 (9.0, 14.0)	201	15.7 (13.6, 18.0)
Adjusted prevalence difference (95% CI; q-value)	−2.0 (−3.9, −0.0; 0.10)		−3.8 (−5.8, −1.7; 0.001)		Referent		2.5 (−0.2, 5.2; 0.14)		7.0 (4.4, 9.5; < 0.001)	

APDs are for “usually or always justified” responses and are adjusted for age, race and ethnicity, gender, education, income, Census division, and rurality. Q-values, also known as FDR-adjusted (or FDR-corrected) p-values, represent the probability that the given difference would be a false discovery; they represent the expected proportion of “false positives” that would be seen among the collection of all differences whose q-values were at or below the given q-value.

**Fig 1:**
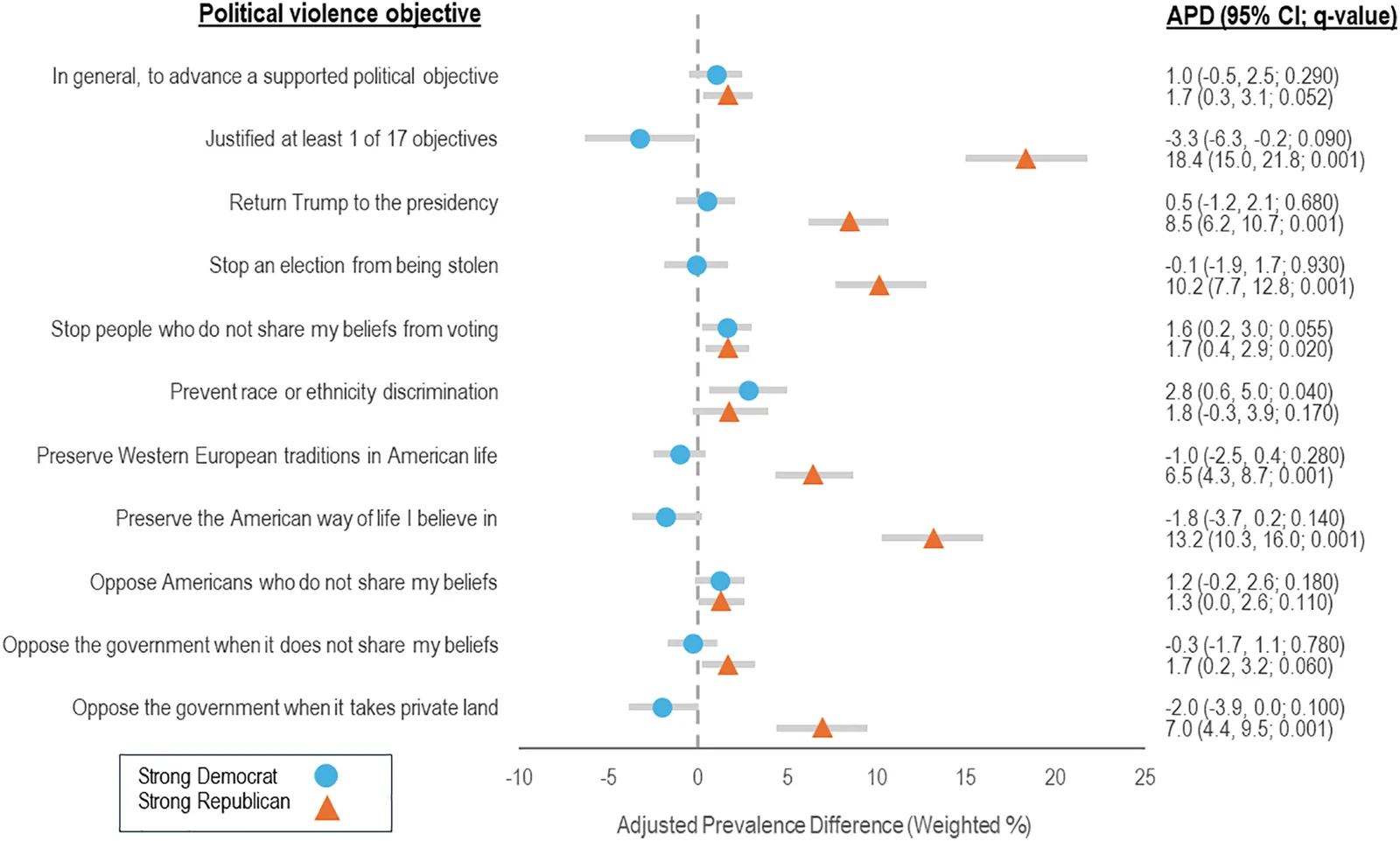
Adjusted prevalence differences for strong Democrats* and strong Republicans* for the view that force or violence is usually or always justified, in general and for 9 specific objectives. * The referent is respondents classified as undecided/independent/other/leans Democrat/leans Republican. APDs are adjusted for age, race and ethnicity, gender, education, income, Census division, and rurality. Q-values, also known as FDR-adjusted (or FDR-corrected) p-values, represent the probability that the given difference would be a false discovery; they represent the expected proportion of “false positives” that would be seen among the collection of all differences whose q-values were at or below the given q-value. .

**Fig 2:**
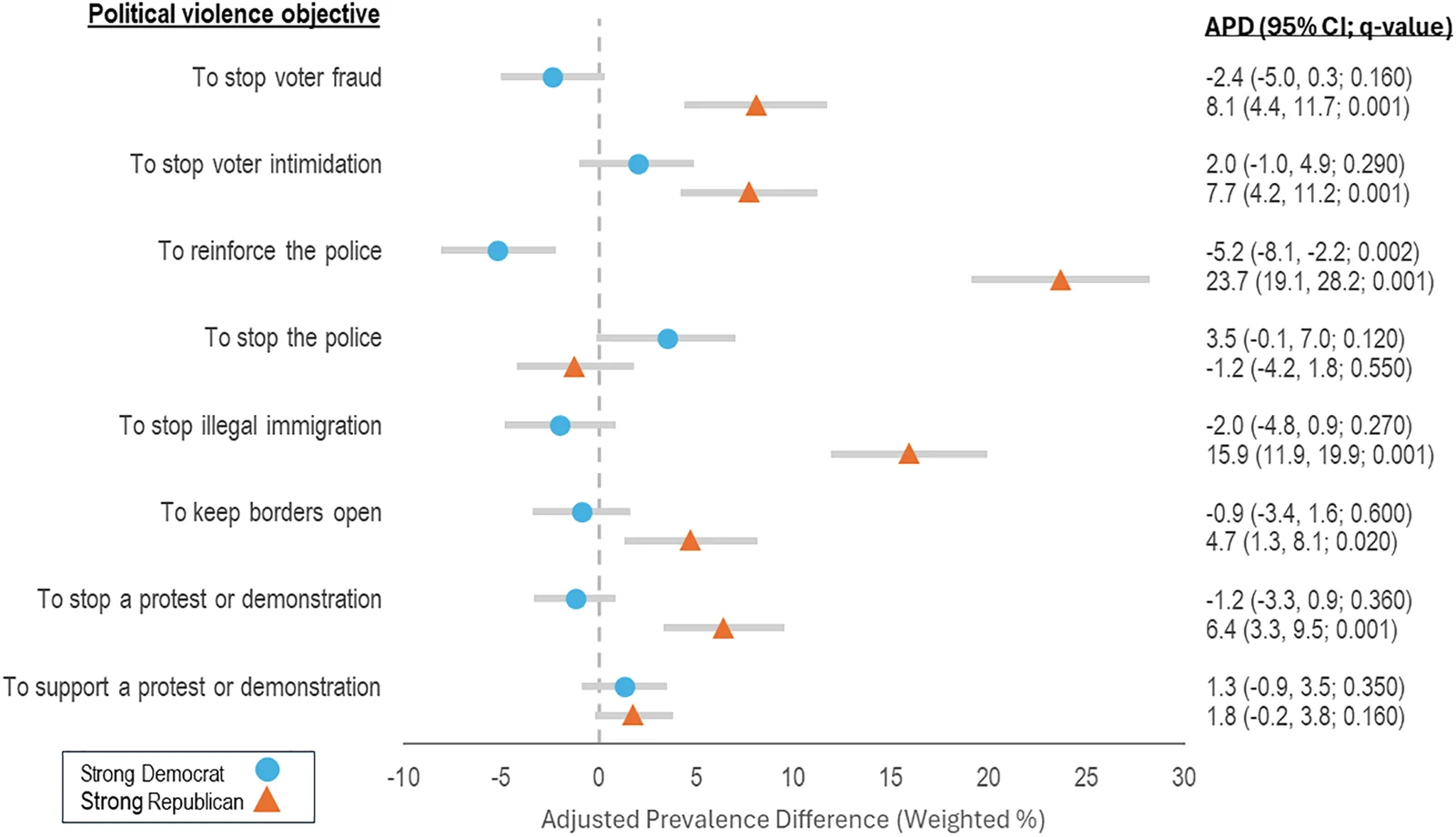
Adjusted prevalence differences for strong Democrats* and strong Republicans* for the view that force or violence is usually or always justified, for 8 additional specific objectives. * The referent is respondents classified as undecided/independent/other/leans Democrat/leans Republican. APDs are adjusted for age, race and ethnicity, gender, education, income, Census division, and rurality. Q-values, also known as FDR-adjusted (or FDR-corrected) p-values, represent the probability that the given difference would be a false discovery; they represent the expected proportion of “false positives” that would be seen among the collection of all differences whose q-values were at or below the given q-value. .

Regardless of their response on the justification for political violence in general, respondents were asked about the justification for political violence to advance 17 specific political objectives (Tables 2 and 3, Figs 1 and 2). Nearly a third of respondents (32.8%, 95% CI 31.7%, 33.9%) considered violence to be usually or always justified to advance at least 1 of these 17 objectives. Strong Republicans were more likely than strong Democrats to see violence as justified for at least 1 objective (aPD 21.7%, 95% CI 17.8%, 25.6%; q < 0.001).

**Table 3:** Party affiliation and justification for political violence for 8 additional specific objectives*.

What do you think about the use of force or violence in the following situations?	Party Affiliation									
	Strong Democrat		Not Very Strong Democrat		Undecided/Independent/Other/Leans		Not Very Strong Republican		Strong Republican	
	Unweighted n	Weighted % (95% CI)	Unweighted n	Weighted % (95% CI)	Unweighted n	Weighted % (95% CI)	Unweighted n	Weighted % (95% CI)	Unweighted n	Weighted % (95% CI)
To stop voter fraud										
Never justified	672	82.5 (79.1, 85.5)	418	75.3 (70.8, 79.3)	1338	75.1 (72.7, 77.3)	326	71.3 (66.5, 75.7)	440	62.5 (58.5, 66.3)
Sometimes justified	73	10.2 (7.9, 13.1)	78	17.7 (14.2, 21.9)	270	15.5 (13.7, 17.6)	94	21.4 (17.4, 25.9)	146	20.4 (17.3, 23.8)
Usually or always justified	49	7.3 (5.4, 9.8)	40	7.0 (5.0, 9.8)	162	9.4 (7.9, 11.1)	35	7.3 (5.0, 10.5)	124	17.2 (14.2, 20.6)
Adjusted prevalence difference (95% CI; q-value)	−2.4 (−5.0, 0.3; 0.16)		−1.8 (−4.7, 1.1; 0.32)		Referent		−0.8 (−3.8, 2.3; 0.71)		8.1 (4.4, 11.7; < 0.001)	
To stop voter intimidation										
Never justified	472	59.1 (55.2, 62.9)	371	66.7 (62.2, 71.0)	1065	62.1 (59.5, 64.7)	307	63.4 (58.3, 68.3)	398	56.2 (52.1, 60.1)
Sometimes justified	244	29.6 (26.2, 33.3)	145	26.3 (22.4, 30.6)	491	28.6 (26.2, 31.1)	124	27.5 (23.2, 32.3)	202	27.2 (23.8, 30.8)
Usually or always justified	90	11.3 (9.0, 13.9)	38	7.0 (4.9, 9.8)	170	9.3 (7.9, 10.9)	36	9.0 (6.0, 13.2)	121	16.7 (13.8, 19.9)
Adjusted prevalence difference (95% CI; q-value)	2.0 (−1.0, 4.9; 0.29)		−2.6 (−5.5, 0.4; 0.17)		Referent		−0.1 (−3.8, 3.7; 0.98)		7.7 (4.2, 11.2; < 0.001)	
To reinforce the police										
Never justified	466	58.9 (55.0, 62.7)	237	47.8 (42.9, 52.7)	753	45.3 (42.7, 48.0)	125	29.1 (24.7, 34.0)	135	19.9 (16.8, 23.4)
Sometimes justified	271	32.3 (28.7, 36.0)	191	38.8 (34.1, 43.7)	734	40.2 (37.7, 42.8)	222	47.8 (42.7, 53.0)	284	39.3 (35.4, 43.3)
Usually or always justified	65	8.8 (6.8, 11.5)	74	13.4 (10.5, 17.0)	280	14.5 (12.8, 16.3)	104	23.1 (18.9, 27.9)	296	40.8 (36.8, 44.9)
Adjusted prevalence difference (95% CI; q-value)	−5.2 (−8.1, −2.2; 0.002)		−1.5 (−5.2, 2.2; 0.54)		Referent		7.5 (2.8, 12.2; 0.006)		23.7 (19.1, 28.2; < 0.001)	
To stop police violence										
Never justified	338	39.8 (36.0, 43.7)	275	44.4 (40.0, 49.0)	811	44.8 (42.2, 47.5)	242	49.2 (44.2, 54.2)	385	53.3 (49.3, 57.3)
Sometimes justified	349	44.1 (40.3, 48.0)	243	42.7 (38.3, 47.3)	715	42.3 (39.7, 45.0)	183	40.0 (35.2, 45.0)	257	36.8 (33.0, 40.8)
Usually or always justified	111	16.1 (13.3, 19.3)	69	12.8 (10.0, 16.4)	203	12.9 (11.1, 14.9)	45	10.7 (7.8, 14.7)	72	9.8 (7.7, 12.4)
Adjusted prevalence difference (95% CI; q-value)	3.5 (−0.1, 7.0; 0.12)		0.2 (−3.5, 3.9; 0.94)		Referent		−0.6 (−4.4, 3.1; 0.82)		−1.2 (−4.2, 1.8; 0.55)	
To stop illegal immigration										
Never justified	652	79.5 (75.9, 82.7)	395	72.3 (67.8, 76.3)	1116	64.1 (61.5, 66.6)	228	47.3 (42.3, 52.4)	228	33.4 (29.7, 37.3)
Sometimes justified	103	13.5 (10.9, 16.4)	108	20.8 (17.2, 24.9)	470	26.1 (23.8, 28.5)	190	40.8 (35.9, 45.8)	285	40.0 (36.2, 44.0)
Usually or always justified	41	7.0 (5.0, 9.8)	33	6.9 (4.8, 9.9)	190	9.8 (8.4, 11.4)	61	11.9 (9.0, 15.7)	193	26.6 (23.2, 30.3)
Adjusted prevalence difference (95% CI; q-value)	−2.0 (−4.8, 0.9; 0.27)		−2.9 (−5.8, 0.1; 0.11)		Referent		1.8 (−1.8, 5.3; 0.44)		15.9 (11.9, 19.9; < 0.001)	
To keep borders open										
Never justified	531	65.2 (61.3, 68.8)	367	64.7 (60.0, 69.1)	1173	67.0 (64.4, 69.5)	287	64.5 (59.5, 69.2)	509	70.1 (66.1, 73.8)
Sometimes justified	225	28.2 (24.8, 31.8)	151	27.8 (23.6, 32.3)	418	25.4 (23.1, 27.9)	118	26.5 (22.3, 31.2)	134	17.9 (15.0, 21.1)
Usually or always justified	47	6.7 (4.9, 9.0)	37	7.6 (5.3, 10.6)	128	7.6 (6.3, 9.2)	37	9.0 (6.4, 12.4)	77	12.0 (9.3, 15.4)
Adjusted prevalence difference (95% CI; q-value)	−0.9 (−3.4, 1.6; 0.60)		−0.3 (−3.4, 2.8; 0.91)		Referent		1.7 (−1.5, 5.0; 0.40)		4.7 (1.3, 8.1; 0.02)	
To stop a protest or demonstration										
Never justified	578	71.4 (67.7, 74.8)	349	63.6 (59.1, 67.9)	1004	59.9 (57.2, 62.5)	185	42.2 (37.1, 47.4)	300	42.9 (39.0, 46.9)
Sometimes justified	188	24.5 (21.3, 27.9)	178	30.5 (26.4, 34.8)	601	34.9 (32.3, 37.5)	224	51.5 (46.2, 56.7)	346	46.6 (42.6, 50.6)
Usually or always justified	27	4.1 (2.7, 6.2)	31	5.9 (4.1, 8.5)	82	5.2 (4.1, 6.7)	28	6.3 (4.2, 9.4)	75	10.6 (8.2, 13.5)
Adjusted prevalence difference (95% CI; q-value)	−1.2 (−3.3, 0.9; 0.36)		0.4 (−2.2, 2.9; 0.86)		Referent		1.7 (−1.1, 4.5; 0.36)		6.4 (3.3, 9.5; < 0.001)	
To support a protest or demonstration										
Never justified	623	74.3 (70.6, 77.8)	415	75.3 (70.8, 79.3)	1442	77.4 (75.1, 79.6)	409	84.1 (80.0, 87.5)	604	83.6 (80.3, 86.5)
Sometimes justified	143	20.0 (16.9, 23.6)	93	18.9 (15.4, 23.0)	304	18.3 (16.3, 20.5)	58	12.2 (9.2, 16.1)	79	12.1 (9.6, 15.1)
Usually or always justified	40	5.7 (4.1, 7.8)	25	5.8 (3.8, 8.8)	64	4.3 (3.2, 5.6)	18	3.7 (2.3, 5.9)	28	4.3 (2.9, 6.3)
Adjusted prevalence difference (95% CI; q-value)	1.3 (−0.9, 3.5; 0.35)		0.8 (−1.9, 3.5; 0.66)		Referent		0.8 (−1.2, 2.8; 0.57)		1.8 (−0.2, 3.8; 0.16)	

* These objectives were paired, with respondents randomized 1:1 to see 1 item in each pair.

APDs are for “usually or always justified” responses and are adjusted for age, race and ethnicity, gender, education, income, Census division, and rurality. Q-values, also known as FDR-adjusted (or FDR-corrected) p-values, represent the probability that the given difference would be a false discovery; they represent the expected proportion of “false positives” that would be seen among the collection of all differences whose q-values were at or below the given q-value.

Republicans also endorsed violence for a greater number of specific political objectives than Democrats did. Nearly half (49.5%, 95% CI 46.6%, 52.3%) of strong Republicans and more than a third (35.3%, 95% CI 31.9%, 38.9%) of not very strong Republicans endorsed violence to advance multiple objectives; approximately one fourth of strong Democrats (26.7%, 95% CI, 24.3%, 29.4%) and not very strong Democrats (24.7%, 95% CI, 21.9%, 27.7%) did so.

For 11 of these 17 specific objectives considered individually, strong Republicans were more likely than strong Democrats to see violence as usually or always justified (Tables 2 and 3, Figs 1 and 2). In 2 cases (preventing discrimination and stopping police violence) strong Democrats were slightly more likely than strong Republicans to see violence as usually or always justified; these differences were not statistically significant.

**Fig 3:**
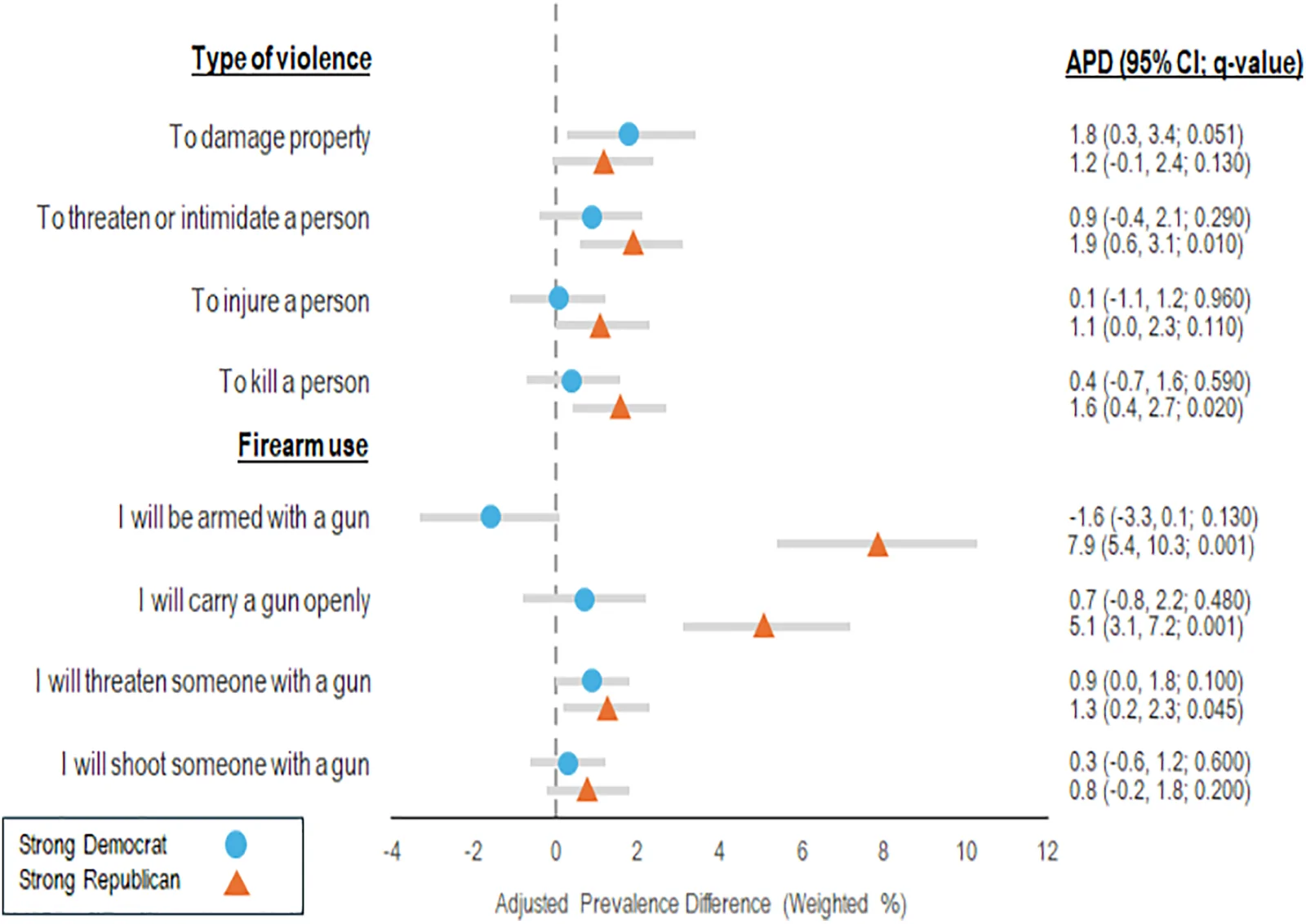
Adjusted prevalence differences for strong Democrats* and strong Republicans* for willingness to engage in political violence†, by type of violence, and for likelihood of future firearm possession and use‡ in a setting where political violence is seen as justified. * The referent is respondents classified as undecided/independent/other/leans Democrat/leans Republican. † The comparison is for the very or completely willing stratum. ‡ The comparison is for the very or extremely likely stratum. APDs are adjusted for age, race and ethnicity, gender, education, income, Census division, and rurality. Q-values, also known as FDR-adjusted (or FDR-corrected) p-values, represent the probability that the given difference would be a false discovery; they represent the expected proportion of “false positives” that would be seen among the collection of all differences whose q-values were at or below the given q-value.

Fewer than 5% of respondents in any party affiliation group reported that they were very or completely willing to damage property or to threaten or intimidate, injure, or kill a person to advance a political objective (Table 4). When compared to the midpoint referent group, strong Republicans were, by small margins, more frequently very or completely willing to “threaten or intimidate a person” and “to kill a person” (Table 4, Fig 3). They did not differ significantly from strong Democrats (threaten a person: aPD 1.0%, 95% CI −0.5%, 2.5%; q = 0.32; kill a person: aPD 1.1%, 95% CI −0.2%, 2.5%; q = 0.19).

**Table 4:** Party affiliation and personal willingness to engage in political violence, by type of violence.

In a situation where you think force or violence is justified to advance an important politicalobjective…How willing would you personally be to use force or violence in each of these ways?	Party Affiliation									
	Strong Democrat		Not Very Strong Democrat		Undecided/Independent/Other/Leans		Not Very Strong Republican		Strong Republican	
	Unweighted n	Weighted % (95% CI)	Unweighted n	Weighted % (95% CI)	Unweighted n	Weighted % (95% CI)	Unweighted n	Weighted % (95% CI)	Unweighted n	Weighted % (95% CI)

To damage property										
Not asked the quesion	1221	73.4 (70.8, 75.9)	830	75.5 (72.6, 78.3)	2443	68.8 (67.0, 70.5)	603	64.7 (61.2, 68.1)	722	50.7 (47.9, 53.6)
Not willing	302	20.1 (17.9, 22.5)	215	19.6 (17.0, 22.4)	892	25.1 (23.5, 26.7)	269	28.7 (25.5, 32.1)	618	42.3 (39.5, 45.2)
Sometimes willing	42	2.9 (2.0, 4.1)	28	2.8 (1.9, 4.1)	102	3.7 (3.0, 4.6)	38	4.6 (3.2, 6.6)	57	4.2 (3.2, 5.6)
Very or completely willing	39	3.6 (2.5, 5.1)	19	2.1 (1.3, 3.4)	69	2.4 (1.9, 3.2)	14	2.0 (1.1, 3.4)	33	2.7 (1.8, 3.9)
Adjusted prevalence difference (95% CI; q-value)	1.8 (0.3, 3.4; 0.051)		0.1 (−1.3, 1.5; 0.91)		Referent		0.3 (−1.0, 1.7; 0.75)		1.2 (−0.1, 2.4; 0.13)	
To threaten or intimidate a person										
Not asked the question	1221	73.4 (70.8, 75.9)	830	75.5 (72.5, 78.2)	2443	68.9 (67.1, 70.6)	603	64.7 (61.2, 68.1)	722	50.7 (47.9, 53.6)
Not willing	318	20.5 (18.3, 22.8)	218	19.7 (17.1, 22.4)	885	24.9 (23.3, 26.5)	267	28.2 (25.1, 31.5)	612	41.8 (39.0, 44.7)
Sometimes willing	41	3.8 (2.7, 5.3)	28	3.0 (2.0, 4.4)	132	4.7 (3.9, 5.7)	45	6.0 (4.3, 8.4)	66	4.9 (3.8, 6.4)
Very or completely willing	24	2.4 (1.5, 3.7)	17	1.9 (1.1, 3.2)	42	1.6 (1.1, 2.2)	9	1.0 (0.5, 2.1)	30	2.6 (1.7, 3.8)
Adjusted prevalence difference (95% CI; q-value)	0.9 (−0.4, 2.1; 0.29)		0.1 (−1.2, 1.3; 0.96)		Referent		0.0 (−0.9, 1.0; 0.96)		1.9 (0.6, 3.1; 0.01)	
To injure a person										
Not asked the question	1221	73.5 (70.9, 75.9)	830	75.5 (72.5, 78.3)	2443	68.9 (67.1, 70.6)	603	64.8 (61.2, 68.2)	722	50.7 (47.8, 53.6)
Not willing	336	22.4 (20.1, 24.8)	223	20.4 (17.9, 23.2)	909	25.8 (24.2, 27.4)	275	29.6 (26.4, 33.0)	623	42.6 (39.8, 45.4)
Sometimes willing	26	2.3 (1.5, 3.7)	19	1.9 (1.1, 3.0)	100	3.6 (2.8, 4.5)	37	4.5 (3.1, 6.3)	59	4.5 (3.4, 6.0)
Very or completely willing	20	1.8 (1.1, 2.9)	20	2.2 (1.4, 3.5)	46	1.8 (1.3, 2.4)	8	1.1 (0.5, 2.5)	27	2.2 (1.5, 3.4)
Adjusted prevalence difference (95% CI; q-value)	0.1 (−1.1, 1.2; 0.96)		0.2 (−1.2, 1.5; 0.86)		Referent		0.2 (−1.1, 1.4; 0.86)		1.1 (−0.0, 2.3; 0.11)	
To kill a person										
Not asked the question	1221	73.4 (70.8, 75.9)	830	75.5 (72.5, 78.2)	2443	68.9 (67.1, 70.6)	603	64.9 (61.4, 68.3)	722	50.7 (47.8, 53.5)
Not willing	344	23.4 (21.1, 25.9)	233	21.3 (18.7, 24.1)	961	27.8 (26.2, 29.6)	290	31.8 (28.5, 35.3)	642	44.4 (41.6, 47.3)
Sometimes willing	15	1.2 (0.6, 2.2)	11	1.3 (0.7, 2.4)	53	1.7 (1.3, 2.4)	18	2.1 (1.3, 3.5)	36	2.4 (1.7, 3.4)
Very or completely willing	23	2.0 (1.3, 3.1)	19	2.0 (1.2, 3.2)	46	1.6 (1.1, 2.2)	11	1.2 (0.6, 2.2)	32	2.5 (1.7, 3.7)
Adjusted prevalence difference (95% CI; q-value)	0.4 (−0.7, 1.6; 0.59)		0.7 (−0.6, 1.9; 0.39)		Referent		0.4 (−0.6, 1.4; 0.59)		1.6 (0.4, 2.7; 0.02)	

APDs are for “very or completely willing” responses and are adjusted for age, race and ethnicity, gender, education, income, Census division, and rurality. Q-values, also known as FDR-adjusted (or FDR-corrected) p-values, represent the probability that the given difference would be a false discovery; they represent the expected proportion of “false positives” that would be seen among the collection of all differences whose q-values were at or below the given q-value.

Similarly, respondents reporting that they were very or completely willing to use force or violence against a person because of that person’s occupation or another social characteristic did not exceed 4% for any party affiliation group (Table 5). Again by small margins, and when compared to the midpoint referent group, strong Republicans were more frequently very or completely willing to engage in violence against “a federal or state government official,” “an election worker, such as a poll worker or vote counter,”, and “a person who does not share your political beliefs.” They again did not differ significantly from strong Democrats (government official: aPD 0.5%, 95% CI −1.1%, 2.2%; q = 0.69; election worker: aPD 0.6%, 95% CI −0.8%, 2.0%; q = 0.53; person not sharing beliefs: aPD 0.7%, 95% CI −0.5%, 1.8%; q = 0.39).

**Table 5:** Party affiliation and personal willingness to engage in political violence, by target of violence.

In a situation where you think force or violence is justified to advance an important politicalobjective…How willing would you personally be to use force or violence against a person because theyare…	Party Affiliation									
	Strong Democrat		Not Very Strong Democrat		Undecided/Independent/Other/Leans		Not Very Strong Republican		Strong Republican	
	Unweighted n	Weighted % (95% CI)	Unweighted n	Weighted % (95% CI)	Unweighted n	Weighted % (95% CI)	Unweighted n	Weighted % (95% CI)	Unweighted n	Weighted % (95% CI)
An elected federal or state government official										
Not asked the quesion	1221	73.5 (70.9, 75.9)	830	75.7 (72.8, 78.5)	2443	69.1 (67.3, 70.8)	603	64.8 (61.2, 68.2)	722	50.8 (48.0, 53.7)
Not willing	327	21.5 (19.3, 23.9)	220	20.3 (17.8, 23.2)	892	25.8 (24.1, 27.4)	286	30.2 (27.0, 33.6)	622	42.9 (40.1, 45.8)
Sometimes willing	26	1.9 (1.3, 2.9)	26	2.5 (1.6, 3.7)	115	3.7 (3.0, 4.5)	23	3.3 (2.1, 5.3)	55	4.0 (2.9, 5.4)
Very or completely willing	29	3.1 (2.1, 4.7)	13	1.5 (0.8, 2.6)	44	1.5 (1.1, 2.1)	11	1.7 (0.9, 3.1)	27	2.3 (1.5, 3.5)
Adjusted prevalence difference (95% CI; q-value)	1.5 (0.1, 2.9; 0.09)		−0.5 (−1.5, 0.7; 0.55)		Referent		1.0 (−0.2, 2.2; 0.21)		2.0 (0.8, 3.3; 0.005)	
An elected local government official										
Not asked the question	1221	73.5 (70.9, 76.0)	830	75.7 (72.7, 78.5)	2443	69.1 (67.3, 70.8)	603	64.9 (61.4, 68.3)	722	50.8 (48.0, 53.7)
Not willing	328	22.0 (19.7, 24.5)	221	19.9 (17.4, 22.7)	908	26.3 (24.6, 28.0)	283	29.6 (26.5, 33.0)	635	44.5 (41.7, 47.4)
Sometimes willing	27	2.0 (1.3, 3.1)	21	2.5 (1.6, 3.9)	100	3.3 (2.6, 4.1)	26	3.4 (2.2, 5.1)	51	3.5 (2.6, 4.7)
Very or completely willing	26	2.5 (1.6, 3.8)	17	1.8 (1.1, 3.1)	41	1.4 (1.0, 2.0)	11	2.1 (1.1, 4.1)	17	1.2 (0.7, 1.9)
Adjusted prevalence difference (95% CI; q-value)	1.2 (−0.1, 2.4; 0.13)		0.2 (−1.0, 1.3; 0.86)		Referent		−1.1 (−0.4, 2.6; 0.27)		0.5 (−0.4, 1.4; 0.36)	
An election worker, such as a poll worker or vote counter										
Not asked the question	1221	73.6 (70.9, 76.0)	830	75.6 (72.6, 78.4)	2443	68.9 (67.1, 70.6)	603	65.0 (61.4, 68.4)	722	50.8 (47.9, 53.6)
Not willing	337	23.0 (20.7, 25.5)	230	20.9 (18.3, 23.7)	972	28.0 (26.3, 29.7)	297	31.6 (28.4, 35.1)	658	45.5 (42.6, 48.3)
Sometimes willing	20	1.4 (0.8, 2.3)	16	1.8 (1.1, 3.1)	53	1.8 (1.3, 2.5)	13	1.9 (0.9, 3.6)	31	2.2 (1.4, 3.4)
Very or completely willing	23	2.1 (1.3, 3.2)	15	1.7 (1.0, 2.9)	33	1.3 (0.9, 1.9)	9	1.5 (0.8, 3.1)	17	1.5 (0.9, 2.7)
Adjusted prevalence difference (95% CI; q-value)	0.9 (−0.3, 2.1; 0.23)		−.02 (−1.3, 1.0; 0.86)		Referent		1.0 (−0.2, 2.3; 0.19)		1.6 (0.4, 2.7; 0.02)	
A public health official										
Not asked the question	1221	73.4 (70.8, 75.9)	830	75.7 (72.7, 78.4)	2443	69.0 (67.3, 70.8)	603	64.9 (61.3, 68.3)	722	50.8 (48.0, 53.7)
Not willing	338	22.9 (20.6, 25.4)	224	20.5 (17.9, 23.3)	939	27.1 (25.5, 28.9)	293	31.2 (28.0, 34.6)	638	44.3 (41.5, 47.2)
Sometimes willing	25	2.0 (1.2, 3.1)	20	2.0 (1.2, 3.3)	71	2.3 (1.8, 3.0)	18	2.3 (1.4, 3.8)	49	3.5 (2.6, 4.8)
Very or completely willing	20	1.7 (1.1, 2.8)	16	1.8 (1.1, 3.0)	40	1.5 (1.0, 2.1)	8	1.6 (0.7, 3.6)	17	1.3 (0.8, 2.1)
Adjusted prevalence difference (95% CI; q-value)	0.4 (−0.7, 1.5; 0.57)		0.2 (−0.9, 1.4; 0.78)		Referent		0.7 (−0.7, 2.0; 0.48)		0.8 (−0.2, 1.7; 0.18)	
A member of the military or National Guard										
Not asked the quesion	1221	73.4 (70.8, 75.9)	830	75.6 (72.6, 78.4)	2443	69.0 (67.2, 70.7)	603	65.0 (61.4, 68.4)	722	50.8 (48.0, 53.7)
Not willing	322	21.5 (19.3, 23.9)	221	20.0 (17.5, 22.8)	916	26.3 (24.6, 28.0)	293	31.4 (28.2, 34.9)	650	45.2 (42.4, 48.1)
Sometimes willing	35	2.4 (1.7, 3.5)	25	2.6 (1.7, 4.0)	92	3.0 (2.4, 3.8)	15	2.2 (1.2, 4.0)	31	2.2 (1.5, 3.1)
Very or completely willing	25	2.6 (1.7, 4.1)	14	1.8 (1.0, 3.1)	46	1.7 (1.2, 2.4)	9	1.5 (0.7, 2.9)	23	1.8 (1.1, 3.0)
Adjusted prevalence difference (95% CI; q-value)	0.9 (−0.4, 2.3; 0.28)		0.0 (−1.2, 1.3; 0.98)		Referent		0.4 (−0.8, 1.6; 0.60)		0.9 (−0.2, 2.1; 0.19)	
A police officer										
Not asked the quesion	1221	73.4 (70.8, 75.9)	830	75.6 (72.6, 78.4)	2443	68.9 (67.1, 70.6)	603	65.2 (61.6, 68.5)	722	50.7 (47.9, 53.6)
Not willing	302	19.8 (17.7, 22.2)	217	19.9 (17.4, 22.8)	909	26.1 (24.5, 27.8)	288	31.0 (27.8, 34.4)	658	45.6 (42.7, 48.4)
Sometimes willing	48	3.7 (2.7, 5.1)	28	3.0 (2.0, 4.4)	89	2.8 (2.2, 3.5)	19	2.5 (1.4, 4.3)	25	1.7 (1.1, 2.5)
Very or completely willing	33	3.0 (2.1, 4.5)	15	1.5 (0.8, 2.6)	59	2.2 (1.7, 2.9)	9	1.4 (0.7, 2.8)	24	2.0 (1.2, 3.3)
Adjusted prevalence difference (95% CI; q-value)	0.9 (−0.5, 2.3; 0.31)		−0.7 (−1.9, 0.5; 0.34)		Referent		−0.1 (−1.2, 1.1; 0.94)		0.9 (−0.3, 2.2; 0.24)	
A person who does not share your race or ethnicity										
Not asked the quesion	1221	73.6 (71.0, 76.0)	830	75.6 (72.6, 78.3)	2443	68.9 (67.1, 70.6)	603	65.0 (61.4, 68.4)	722	50.9 (48.0, 53.7)
Not willing	335	22.4 (20.1, 24.9)	227	20.3 (17.8, 23.1)	972	27.8 (26.2, 29.5)	294	31.5 (28.2, 34.9)	657	45.7 (42.8, 48.6)
Sometimes willing	24	2.1 (1.3, 3.3)	19	2.4 (1.5, 3.8)	50	1.8 (1.3, 2.5)	17	2.7 (1.5, 4.7)	28	1.9 (1.3, 2.9)
Very or completely willing	20	1.9 (1.2, 3.2)	16	1.7 (1.0, 2.9)	35	1.5 (1.0, 2.1)	6	0.9 (0.4, 2.1)	18	1.5 (0.9, 2.6)
Adjusted prevalence difference (95% CI; q-value)	0.2 (−0.9, 1.4; 0.79)		−0.2 (−1.4, 0.9; 0.78)		Referent		−0.0 (−0.9, 0.9; 0.99)		1.1 (0.0, 2.1; 0.10)	
A person who does not share your religion										
Not asked the quesion	1221	73.6 (71.0, 76.1)	830	75.7 (72.7, 78.4)	2443	69.0 (67.2, 70.7)	603	64.9 (61.3, 68.3)	722	50.8 (48.0, 53.7)
Not willing	337	22.7 (20.4, 25.2)	222	19.9 (17.4, 22.7)	965	27.9 (26.2, 29.6)	303	33.2 (29.8, 36.7)	673	46.8 (43.9, 49.7)
Sometimes willing	22	1.7 (1.1, 2.7)	16	1.9 (1.1, 3.2)	52	1.9 (1.4, 2.6)	10	1.2 (0.6, 2.3)	19	1.5 (0.9, 2.6)
Very or completely willing	21	2.1 (1.3, 3.3)	22	2.5 (1.6, 3.9)	33	1.2 (0.8, 1.8)	6	0.8 (0.3, 1.8)	12	0.9 (0.4, 1.7)
Adjusted prevalence difference (95% CI; q-value)	0.6 (−0.6, 1.6; 0.43)		0.9 (−0.4, 2.1; 0.29)		Referent		0.1 (−0.7, 1.0; 0.83)		0.7 (−0.1, 1.5; 0.19)	
A person who does not share your political beliefs										
Not asked the quesion	1221	73.6 (70.9, 76.0)	830	75.6 (72.6, 78.3)	2443	68.9 (67.2, 70.7)	603	65.0 (61.4, 68.3)	722	50.9 (48.0, 53.8)
Not willing	326	21.5 (19.2, 23.9)	229	20.9 (18.3, 23.7)	960	27.8 (26.1, 29.5)	296	31.9 (28.6, 35.4)	653	45.4 (42.6, 48.3)
Sometimes willing	38	3.4 (2.3, 4.8)	16	1.8 (1.0, 2.9)	68	2.2 (1.7, 2.9)	15	2.2 (1.2, 4.0)	30	2.2 (1.4, 3.2)
Very or completely willing	17	1.6 (1.0, 2.6)	17	1.8 (1.1, 2.9)	29	1.1 (0.7, 1.7)	7	1.0 (0.5, 2.2)	19	1.5 (0.9, 2.5)
Adjusted prevalence difference (95% CI; q-value)	0.5 (−0.5, 1.4; 0.45)		0.7 (−0.4, 1.7; 0.36)		Referent		0.3 (−0.6, 1.2; 0.66)		1.1 (0.2, 2.1; 0.04)	

APDs are for “very or completely willing” responses and are adjusted for age, race and ethnicity, gender, education, income, Census division, and rurality. Q-values, also known as FDR-adjusted (or FDR-corrected) p-values, represent the probability that the given difference would be a false discovery; they represent the expected proportion of “false positives” that would be seen among the collection of all differences whose q-values were at or below the given q-value.

### Possession and use of firearms

Finally, all 8,620 respondents were asked, regardless of their position on political violence or firearm ownership status, to predict the likelihood of their future use of a firearm “in a situation where you think force or violence is justified to advance an important political objective” (Table 6, Fig 3). Strong Republicans, more frequently than strong Democrats, thought it very or extremely likely that, in such a situation, “I will be armed with a gun” (aPD 9.5%, 95% CI 6.9%, 12.1%; q < 0.001) and that “I will carry a gun openly, so that people know I am armed” (aPD 4.4%, 95% CI 2.2%, 6.7%; q < 0.001). Fewer than 2% of respondents thought it very or extremely likely that “I will threaten someone with a gun” or that “I will shoot someone with a gun.” Except for a small and marginally significant increase in predicted threats with guns among strong Republicans as compared to the midpoint referent group, there was no statistically significant variation across party affiliation.

**Table 6:** Party affiliation and likelihood of firearm possession and use when political violence is perceived as justified.

Thinking now about the future and all the changes it might bring, how likely is it that you will use a gun in any of the following ways in the next few years—in a situation where you think force or violence is justified to advance an important political objective?	Party Affiliation									
	Strong Democrat		Not Very Strong Democrat		Undecided/Independent/Other/Leans		Not Very Strong Republican		Strong Republican	
	Unweighted n	Weighted % (95% CI)	Unweighted n	Weighted % (95% CI)	Unweighted n	Weighted % (95% CI)	Unweighted n	Weighted % (95% CI)	Unweighted n	Weighted % (95% CI)
I will be armed with a gun.										
Not likely	1463	89.1 (87.0, 90.9)	950	85.5 (82.9, 87.8)	2886	81.1 (79.5, 82.6)	727	76.5 (73.1, 79.6)	1057	71.5 (68.7, 74.1)
Somewhat likely	86	6.3 (4.9, 7.9)	91	9.6 (7.7, 11.8)	352	11.4 (10.1, 12.7)	123	15.1 (12.6, 18.2)	181	13.9 (12.0, 16.1)
Very or extremely likely	53	4.7 (3.4, 6.3)	45	4.9 (3.6, 6.6)	237	7.5 (6.6, 8.6)	64	8.3 (6.5, 10.7)	173	14.6 (12.5, 17.0)
Adjusted prevalence difference (95% CI; q-value)	−1.6 (−3.3, 0.1; 0.13)		−2.3 (−4.1, 0.5; 0.04)		Referent		1.7 (−0.7, 4.0; 0.27)		7.9 (5.4, 10.3; < 0.001)	
I will carry a gun openly, so that people know I am armed.										
Not likely	1532	93.8 (92.0, 95.3)	1016	92.9 (90.9, 94.5)	3174	90.4 (89.1, 91.5)	831	88.3 (85.4, 90.8)	1202	83.0 (80.5, 85.3)
Somewhat likely	36	2.6 (1.8, 3.8)	40	4.2 (3.0, 5.9)	182	5.9 (5.0, 6.9)	61	8.6 (6.5, 11.2)	116	8.8 (7.2, 10.6)
Very or extremely likely	35	3.5 (2.4, 5.1)	28	2.9 (1.9, 4.2)	114	3.7 (3.1, 4.6)	21	3.1 (1.9, 4.9)	91	8.2 (6.5, 10.3)
Adjusted prevalence difference (95% CI; q-value)	0.7 (−0.8, 2.2; 0.48)		−0.6 (−1.9, 0.8; 0.52)		Referent		−0.0 (−1.7, 1.6; 0.98)		5.1 (3.1, 7.2; < 0.001)	
I will threaten someone with a gun.										
Not likely	1573	97.5 (96.3, 98.4)	1056	96.5 (94.9, 97.6)	3406	97.8 (97.1, 98.3)	906	98.5 (97.2, 99.2)	1386	97.7 (96.3, 98.6)
Somewhat likely	12	1.1 (0.5, 2.1)	19	2.1 (1.4, 3.4)	42	1.5 (1.0, 2.1)	10	1.2 (0.6, 2.4)	10	0.8 (0.4, 1.6)
Very or extremely likely	16	1.4 (0.8, 2.4)	10	1.4 (0.7, 2.6)	21	0.7 (0.4, 1.1)	1	0.2 (0.0, 1.7)	13	1.5 (0.8, 2.8)
Adjusted prevalence difference (95% CI; q-value)	0.9 (0.0, 1.8; 0.10)		0.6 (−0.4, 1.6; 0.34)		Referent		−0.1 (−0.7, 0.5; 0.86)		1.3 (0.2, 2.3; 0.045)	
I will shoot someone with a gun.										
Not likely	1558	96.5 (95.0, 97.5)	1043	95.4 (93.6, 96.7)	3358	96.0 (95.1, 96.8)	894	97.1 (95.4, 98.1)	1358	95.4 (93.8, 96.6)
Somewhat likely	26	2.2 (1.4, 3.5)	28	3.0 (2.0, 4.5)	84	2.9 (2.3, 3.8)	20	2.7 (1.7, 4.3)	39	3.3 (2.3, 4.5)
Very or extremely likely	15	1.3 (0.7, 2.3)	13	1.6 (0.9, 2.9)	33	1.0 (0.7, 1.5)	2	0.3 (0.1, 1.0)	13	1.3 (0.7, 2.6)
Adjusted prevalence difference (95% CI; q-value)	0.3 (−0.6, 1.2; 0.60)		0.4 (−0.6, 1.4; 0.54)		Referent		−0.4 (−0.9, 0.2; 0.31)		0.8 (−0.2, 1.8; 0.20)	

APDs are for “very or extremely likely” responses and are adjusted for age, race and ethnicity, gender, education, income, Census division, and rurality. Q-values, also known as FDR-adjusted (or FDR-corrected) p-values, represent the probability that the given difference would be a false discovery; they represent the expected proportion of “false positives” that would be seen among the collection of all differences whose q-values were at or below the given q-value.

### Findings for political ideology

Patterns of variation seen with political ideology (ranging from extremely conservative to extremely liberal) largely reflected those seen with party affiliation (ranging from strong Republican to strong Democrat); see S9-S18 Tables in S1 File. For example, extreme conservatives were more likely than extreme liberals to agree strongly or very strongly that “having a strong leader for America is more important than having a democracy,” to endorse racist and conspiratorial beliefs, and to predict civil war. They more frequently endorsed political violence to advance at least 1 of 17 specific objectives and to advance 8 of those 17 objectives considered individually (aPDs are available from the authors).

### Sensitivity analysis

Sociodemographic characteristics of attentive and inattentive respondents are given in S19 Table in S1 File. Compared to attentive respondents, the 447 respondents who failed the attentiveness check had a substantially (but statistically non-significantly) higher prevalence of prior arrest for violent offenses (inattentives, 34.0%, 95% CI, 22.7%, 47.4%; attentives, 23.0%, 95% CI, 20.3%, 26.0%) (q = 0.077), a significantly higher prevalence of prior arrest for firearm-related offenses (inattentives, 11.9%, 95% CI, 5.9%, 22.7%; attentives, 4.2%, 95% CI, 3.1%, 5.7%) (q = 0.017) and, among those with prior firearm-related arrests, a marginally significant two-fold higher prevalence of arrests for firearm-related violent offenses (inattentives, 67.7%, 95% CI, 32.4%, 90.2%; attentives, 31.1%, 95% CI, 18.7%, 47.0%) (q = 0.054) (S20 Table in S1 File).

Results excluding the 447 inattentives were similar to those presented here. Modeled prevalence differences for the whole sample and with inattentives excluded showed a frequency difference of >1 and >2 percentage points 18.2% and 3.5% of the time, respectively, for party affiliation and 31.8% and 12.5% of the time, respectively, for political ideology.

## Discussion

This study proceeded from the position that political violence and its potential antecedents can usefully be examined from a public health perspective, as is true of other forms of violence. With concern for the stability of democracy and for outbreaks of political violence rising rapidly in the US [1–14], we sought to learn whether beliefs about democracy and social issues, support for political violence, and personal willingness to engage in political violence varied across the spectrum of party affiliation and political ideology.

The beliefs that it is important for the US to remain a democracy and that democracy is the best form of government are widely shared across party affiliations and political ideologies, but other findings are much less encouraging. While most respondents believe that American democracy faces a serious threat, many respondents across the political spectrum and most extreme liberals believe strongly or very strongly that American democracy serves only the interests of the wealthy and powerful. Support for expressions of authoritarianism, such as “having a strong leader for America is more important than having a democracy,” is concerningly high among strong Republicans and extreme conservatives.

There are stark differences across the political spectrum on matters involving race and ethnicity and the distribution of power in the US. High prevalences of strong or very strong belief in the QAnon and great replacement delusions among strong Republicans and extreme conservatives are particularly striking. Both have been linked to violence in specific cases [52–54] and are plausible antecedents to political violence.

There are both concerning and hopeful findings on political violence. It is concerning that support for general statements about the need for violence under current or plausible future conditions and endorsement of political violence are frequently quite common, more so here among Republicans and conservatives than Democrats and liberals. But there is hope in the findings that most respondents rejected political violence altogether, and that of those who considered violence usually or always justified to advance at least 1 political objective, relatively few were more than somewhat willing to commit such violence themselves.

These hopeful findings come with caveats, however. First, endorsement of violence by those who are unwilling to commit violence themselves may help create a climate of acceptance and promote violence by others [55]. Second, as there were an estimated 258 million adults in the US as of July 1, 2021 [56], small percentages of respondents in this sample represent large numbers of people. Third, the perception that partisan differences create an existential threat has become widespread. In the latter half of 2022, 81% of Democrats and 79% of Republicans agreed that the other party’s “agenda poses a threat that if not stopped will destroy America as we know it” [57]. Most Democrats (58%) and Republicans (56%) agreed that members of the other party are “enemies—that is, if they win, your life or your entire way of life may be threatened” [58].

To the extent that these topics have been explored previously, our findings are largely concordant with prior work [1–8,26–31,59–63]. In the aggregate, this body of work suggests a real possibility that large-scale political violence will emerge in the US in the near future [64,65].

Describing how support for and willingness to engage in political violence is distributed across the population is important for understanding the problem and developing effective prevention efforts. Further studies from this survey will examine other potential risk factors and seek to develop predictive models of support for political violence. The Center for Prevention Programs and Partnerships [66], a targeted violence prevention program of the Department of Homeland Security, was developed following “principles for violence prevention” articulated by the Centers for Disease Control and Prevention. Its operations through 2021 have, however, been criticized as “plagued by fundamental flaws, bias, and a reliance on ineffective methods” [67]. Fortunately, new research and analysis is identifying potentially efficacious interventions that could be adapted to specific high-risk groups or implemented at the population level [12,32,33,68]. Given the findings of this study, consideration might be given to implementing such interventions in partnership with Republican or conservative thought leaders and organizations.

### Limitations

While the study assessed respondents’ support for violence to advance specific objectives, it did not specify the circumstances in which that support might (or might not) be given. For example, support for violence “to preserve the American way of life I believe in” might increase substantially if the US were under attack by the armed forces of another country. More broadly, the study did not proceed from an a priori judgement of the moral status of political violence—that it is innately and always “good” or “bad.” It is worth noting, however, that support for political violence in this cohort has been highest among those who strongly agree with expressions of racism, hostile sexism and other forms of hatred, fear and enmity toward others [69] and among those who strongly approve of organizations such as the Proud Boys and Oath Keepers and social movements such as the white supremacy movement and Christian nationalism [70].

Strengths associated with use of a sample from KnowledgePanel were described in the Methods section; several limitations exist as well. Persons without addresses are not recruited into KnowledgePanel. The participation rate was somewhat lower than expected. Crosstabulations frequently produce response counts of <100, and weighted prevalences for some important outcomes are below 5%. The large study sample results in relatively narrow confidence intervals in these cases, however.

The findings are cross-sectional, and findings for 2023 or 2024 might differ. For example, larger partisan or ideological differences might be expected in 2024, a presidential election year that involved the attempted assassination of a major party candidate for the presidency. Widely publicized mass shootings occurred in Buffalo, NY and Uvalde, TX while the survey was in the field. The Buffalo shooting is understood to have been a race-related hate crime motivated by the great-replacement belief [53,54] and may have affected respondents’ views on race, violence, and replacement. Russia’s war against Ukraine may have influenced responses on violence and democracy.

Other limitations are specific to this analysis. The original construction of the party affiliation variable did not include a true undecided/no affiliation stratum large enough to serve as a referent. Prevalences of support for political violence to advance 1 or more of 17 specific political objectives are in part a function of the specific objectives presented; a different list might have produced different results. There were inattentive respondents, but they accounted for only 5.2% of respondents by our measure and excluding them did not affect the results. In our sample, inattentives gave other responses suggesting a higher risk for violence generally. We conclude that while including inattentives may increase estimates of support for political violence, it may also decrease bias in those estimates; others may disagree [8].

The findings are also subject to social desirability bias, though that is less a factor in self-administered surveys such as this than in interview surveys [42]. In particular, support for and willingness to engage in political violence might be perceived as stigmatized; our survey may underestimate prevalences for them. We did not ask respondents to report acts of political violence; such acts would have been crimes, given our definition, and likely subject to substantial underreporting. Nor did we solicit specific information on what gives rise to support for political violence, or on how that support or its causes might best be addressed in prevention efforts. These remain subjects for future research.

## Conclusions

Findings from this large, nationally representative survey suggest support for and willingness to engage in political violence are most prevalent at the political and ideological extremes and generally more common among Republicans and conservatives than Democrats and liberals. As these associations become better understood, risk-based interventions are likely to become more efficacious, particularly if coupled with population-based interventions on the factors that give rise to violence generally. Most respondents rejected political violence outright, in general and to advance any specific political objective, and most of those who endorsed it were unwilling to engage in it themselves.

## Declarations and acknowledgments

### Consent to participate

Introductory text to the questionnaire as seen by participants included this statement:

As with all KnowledgePanel surveys, your participation is entirely voluntary, and your responses will be kept confidential and anonymous. You will not be individually identified, and your de-identified responses will only be used for qualified research purposes. You may skip any question at any time.

If you have any questions about this survey, you may contact the research team by calling (916) 734-3539. This study has been approved by the Institutional Review Board of the University of California, Davis. If you have any questions about your rights as a participant in this study, you may contact the University of California, Davis, Institutional Review Board at (916) 703-9151. If you have questions about your rights as a research subject or are dissatisfied at any time with any aspect of the survey, you may also contact KnowledgePanel panel member support at (800) 782-6899.

By continuing, you are agreeing to participate in this study.

## Supporting information

S1 File Supplement. Includes survey items that were included in this analysis, and 20 supplementary tables.
(ZIP)

S2 File Appendix. (DOCX)

## References

[1] Cox DA. After the ballots are counted: conspiracies, political violence, and American exceptionalism: findings from the January 2021 American Perspectives Survey. American Enterprise Institute, Survey Center on American Life; 2021. https://www.americansurveycenter.org/research/after-the-ballots-are-counted-conspiracies-political-violence-and-american-exceptionalism/

[2] Public Religion Research Institute. The persistence of Q-Anon in the post-Trump era: an analysis of who believes the conspiracies. 2022. https://www.prri.org/research/the-persistence-of-qanon-in-the-post-trump-era-an-analysis-of-who-believes-the-conspiracies/

[3] Economist/YouGov Poll, December 31, 2022 – January 3, 2023. 2023. Accessed 2023 January 3. https://today.yougov.com/topics/politics/explore/topic/The_Economist_YouGov_polls

[4] Safarpour AC, Druckman J, Lazer D, Trujillo KL, Shere A, Baum MA. Americans’ views on violence against the government. COVID-19 Consortium for Understanding the Public’s Policy Preferences Across States; 2022. https://www.covidstates.org/reports/americans-views-on-violence-against-the-government

[5] Bright Line Watch. American democracy on the eve of the 2020 election. 2020. http://brightlinewatch.org/american-democracy-on-the-eve-of-the-2020-election/

[6] Bright Line Watch. Tempered expectations and hardened divisions a year into the Biden presidency. 2021. http://brightlinewatch.org/tempered-expectations-and-hardened-divisions-a-year-into-the-biden-presidency/

[7] Kalmoe NP, Mason L. Radical American partisanship. Chicago: University of Chicago Press; 2022.

[8] Westwood SJ, Grimmer J, Tyler M, Nall C. Current research overstates American support for political violence. Proceedings of the National Academy of Sciences. 2022;119(12):e2116870119. doi: 10.1073/pnas.2116870119 PMC8944847 35302889

[9] Feuer A, Haberman M. Hard right stokes outrage after search of Mar-a-Lago. New York Times. 2022.

[10] Bump P. Trumpworld walks a line between predicting violence and threatening it. Washington Post. 2022.

[11] Kleinfeld R. The rise of political violence in the United States. Journal of Democracy. 2021;32(4):160–76. doi: 10.1353/jod.2021.0059

[12] Walter BF. How civil wars start and how to stop them. New York: Crown. 2022.

[13] Bulletin: summary of the terrorism threat to the United States. Department of Homeland Security; 2022. https://www.dhs.gov/national-terrorism-advisory-system

[14] House Select Committee to Investigate the January 6th Attack on the United States Capitol. Select January 6th Committee final report and supporting materials collection. 2022. https://www.govinfo.gov/collection/january-6th-committee-final-report?path=/GPO/January%206th%20Committee%20Final%20Report%20and%20Supporting%20Materials%20Collection

[15] Armed Conflict Location and Event Data Project. ACLED definitions of political violence and protest. 2019. https://acleddata.com/acleddatanew/wp-content/uploads/2021/11/ACLED_Event-Definitions_v1_April-2019.pdf

[16] Applebome P. Conversations/David Satcher; C.D.C.’s new chief worries as much about bullets as about bacteria. New York Times. 1993.

[17] Sousa CA. Political violence, collective functioning and health: a review of the literature. Med Confl Surviv. 2013;29(3):169–97. doi: 10.1080/13623699.2013.813109 24133929 PMC3801099

[18] Davenport C, Nygård H, Fjelde H, Armstrong D. The consequences of contention: understanding the aftereffects of political conflict and violence. Annu Rev Polit Sci. 2019;22:361–77.

[19] Wintemute GJ, Crawford A, Robinson SL, Tomsich EA, Reeping PM, Schleimer JP, et al. Firearm ownership and support for political violence in the United States. JAMA Netw Open. 2024;7(4):e243623. doi: 10.1001/jamanetworkopen.2024.3623 38592725 PMC11004826

[20] Decker MR, Wilcox HC, Holliday CN, Webster DW. An integrated public health approach to interpersonal violence and suicide prevention and response. Public Health Rep. 2018;133(1_suppl):65S-79S. doi: 10.1177/0033354918800019 30426878 PMC6243443

[21] Haddon W Jr. On the escape of tigers: an ecologic note. Am J Public Health Nations Health. 1970;60(12):2229–34. doi: 10.2105/ajph.60.12.2229-b 5530409 PMC1349282

[22] Haddon W Jr. Advances in the epidemiology of injuries as a basis for public policy. Public Health Rep. 1980;95(5):411–21. 7422807 PMC1422748

[23] Rivara FP, Richmond TS, Hargarten S, Branas CC, Rowhani-Rahbar A, Webster D, et al. Toward a Safer World by 2040: the JAMA summit report on reducing firearm violence and harms. JAMA. 2025;334(24):2208–19. doi: 10.1001/jama.2025.18076 41182880

[24] Division of Unintentional Injury Prevention. Motor-vehicle safety: a 20th century public health achievement. MMWR. 1999;48(10):369–74. 10369577

[25] Hemenway D, Lee LK. Lesson from the continuing 21st century motor vehicle success. Inj Prev. 2022;28(5):480–2. doi: 10.1136/ip-2022-044635 35790347

[26] Wintemute GJ, Robinson SL, Crawford A, Tancredi D, Schleimer JP, Tomsich EA, et al. Views of democracy and society and support for political violence in the USA: findings from a nationally representative survey. Inj Epidemiol. 2023;10(1):45. doi: 10.1186/s40621-023-00456-3 37770994 PMC10540371

[27] Wintemute GJ, Robinson SL, Tomsich EA, Tancredi DJ. MAGA Republicans’ views of American democracy and society and support for political violence in the United States: findings from a nationwide population-representative survey. PLoS One. 2024;19(1):e0295747. doi: 10.1371/journal.pone.0295747 38170700 PMC10763974

[28] Lambert L. Here’s where Republicans and Democrats stand on using violence to advance political goals. Fortune. 2021.

[29] Topazian RJ, McGinty EE, Han H, Levine AS, Anderson KE, Presskreischer R, et al. US adults’ beliefs about harassing or threatening public health officials during the COVID-19 pandemic. JAMA Netw Open. 2022;5(7):e2223491. doi: 10.1001/jamanetworkopen.2022.23491 35904784 PMC9338413

[30] Jones RP, Jackson N, Orcés D, Huff I, Holcomb T. Competing visions of America: an evolving identity or a culture under attack?. Washington, DC: Public Religion Research Institute; 2021. https://www.prri.org/research/competing-visions-of-america-an-evolving-identity-or-a-culture-under-attack/

[31] Braley A, Lenz GS, Adjoday D, Rahnama H, Pendland A. The subversion dilemma: why voters who cherish democracy participate in democratic backsliding. 2022. 10.1038/s41562-023-01594-w 37217740

[32] Mernyk JS, Pink SL, Druckman JN, Willer R. Correcting inaccurate metaperceptions reduces Americans’ support for partisan violence. 2022. doi: 10.31219/osf.io/8ftsx PMC9169855 35412915

[33] Voelkel JG, Stagnaro MN, Chu J, Pink S, Mernyk JS, Redekopp C. Megastudy identifying successful interventions to strengthen Americans’ democratic attitudes. 2022. https://www.strengtheningdemocracychallenge.org/paper

[34] Ipsos. https://www.ipsos.com/en

[35] American Association for Public Opinion Research. Code of professional ethics and practices. 2021. https://www.aapor.org/Standards-Ethics/AAPOR-Code-of-Ethics.aspx

[36] Kravitz-Wirtz N, Aubel A, Schleimer J, Pallin R, Wintemute G. Public concern about violence, firearms, and the covid-19 pandemic in California. JAMA Netw Open. 2021;4(1):e2033484. doi: 10.1001/jamanetworkopen.2020.33484 33394004 PMC7783542

[37] Wintemute GJ, Aubel AJ, Pallin R, Schleimer JP, Kravitz-Wirtz N. Experiences of violence in daily life among adults in California: a population-representative survey. Inj Epidemiol. 2022;9(1):1. doi: 10.1186/s40621-021-00367-1 34980276 PMC8721630

[38] Schleimer JP, Kravitz-Wirtz N, Pallin R, Charbonneau AK, Buggs SA, Wintemute GJ. Firearm ownership in California: a latent class analysis. Inj Prev. 2020;26(5):456–62. doi: 10.1136/injuryprev-2019-043412 31601624

[39] Miller M, Zhang W, Azrael D. Firearm purchasing during the COVID-19 pandemic: results from the 2021 national firearms survey. Ann Intern Med. 2022;175(2):219–25. doi: 10.7326/M21-3423 34928699 PMC8697522

[40] Miller M, Azrael D. Firearm storage in US households with children: findings from the 2021 national firearm survey. JAMA Netw Open. 2022;5(2):e2148823. doi: 10.1001/jamanetworkopen.2021.48823 35191973 PMC8864510

[41] Salhi C, Azrael D, Miller M. Patterns of gun owner beliefs about firearm risk in relation to firearm storage: a latent class analysis using the 2019 National Firearms Survey. Inj Prev. 2020. doi: injuryprev-2019-043624 10.1136/injuryprev-2019-043624 32665253

[42] Ipsos. KnowledgePanel® sample representativeness & related topics: a methodology memorandum. 2020.

[43] Ipsos. KnowledgePanel®: a methodological overview. Ipsos; 2020. https://www.ipsos.com/sites/default/files/ipsosknowledgepanelmethodology.pdf

[44] Ipsos. KnowledgePanel sampling and weighting methodology. 2023. https://www.ipsos.com/sites/default/files/kpsamplingandweighting.pdf

[45] United States Census Bureau. National Population by Characteristics: 2020-2021. 2022. https://www.census.gov/data/tables/time-series/demo/popest/2020s-national-detail.html

[46] American National Election Studies. American national election studies. https://electionstudies.org/

[47] Malhotra N, Kuo AG. Emotions as moderators of information cue use citizen attitudes toward Hurricane Katrina. Am Politics Res. 2009;37:301–26.

[48] Bracke S, Aguilar LMH. The politics of replacement: from “race suicide” to the “Great Replacement.”. In: Bracke S, Aguilar LMH, editors. The politics of replacement: demographic fears, conspiracy theories, and race wars. Milton Park: Routledge; 2023.

[49] Chyung SYY, Roberts K, Swanson I, Hankinson A. Evidence-based survey design: the use of a midpoint on the likert scale. Perf Improv. 2017;56(10):15–23. doi: 10.1002/pfi.21727

[50] Benjamini Y, Hochberg Y. Controlling the false discovery rate: a practical and powerful approach to multiple testing. J R Statist Soc B. 1995;57(1):289–300.

[51] Storey JD. The positive false discovery rate: a Bayesian interpretation and the q-value. Ann Statist. 2003;31(6):2013–35.

[52] Federal Bureau of Investigation. Anti-government, identity based, and fringe political conspiracy theories very likely motivate some domestic extremists to commit criminal, sometimes violent activity. 2019. https://www.justsecurity.org/wp-content/uploads/2019/08/420379775-fbi-conspiracy-theories-domestic-extremism.pdf

[53] Stanley-Becker I, Harwell D. Buffalo suspect allegedly inspired by racist theory fueling global carnage. Washington Post. 2022.

[54] Popli N. How the ‘great replacement theory’ has fueled racist violence. Time Magazine. 2022.

[55] Byman DL. How hateful rhetoric connects to real-world violence. 2021.

[56] United States Census Bureau. National Population by Characteristics: 2020-2021. 2022. https://www.census.gov/data/tables/time-series/demo/popest/2020s-national-detail.html

[57] Murray M. “Anger on their minds”: NBC News poll finds sky-high interest and polarization ahead of midterms. 2022.

[58] Bump P. Democrats have joined Republicans in calling their opponents ‘enemies’. Washington Post. 2022.

[59] Survey Center on American Life. January 2021 American Perspectives Survey topline questionnaire. Survey Center on American Life; 2021. https://www.americansurveycenter.org/wp-content/uploads/2021/03/January-2021-APS-Topline-Questionnaire.pdf

[60] Zogby. Will the US have another civil war?. 2021. https://zogbyanalytics.com/news/997-the-zogby-poll-will-the-us-have-another-civil-war

[61] Pew Research Center. Americans see advantages and challenges in country’s growing racial and ethnic diversity. 2019. https://www.pewresearch.org/social-trends/2019/05/08/americans-see-advantages-and-challenges-in-countrys-growing-racial-and-ethnic-diversity/

[62] IFYC – PRRI survey on religion & COVID-19 vaccine trust. 2021. https://www.prri.org/wp-content/uploads/2021/05/Topline-IFYC-PRRI-Survey-on-Religion-and-COVID-19-Vaccine-Trust-v2_final.pdf

[63] Romano A. Poll: 61% of Trump voters agree with idea behind ‘great replacement’ conspiracy theory. 2022. https://news.yahoo.com/hed-poll-61-of-trump-voters-agree-with-idea-behind-great-replacement-conspiracy-theory-090004062.html

[64] Sargent G. An expert in political violence urgently warns: the worst is coming. Washington Post. 2022.

[65] Ottesen KK. They are preparing for war: an expert on civil wars discusses where political extremists are taking this country. Washington Post. 2022.

[66] Department of Homeland Security, Center for Prevention Programs and Partnerships. Center for prevention programs and partnerships. 2023.

[67] Reynolds S, Sanchez G. DHS ‘Violence prevention’ grants continue to harm marginalized communities. 2023. https://www.brennancenter.org/our-work/analysis-opinion/dhs-violence-prevention-grants-continue-harm-marginalized-communities

[68] Kleinfeld R. Five strategies to support U.S. democracy. 2022. https://carnegieendowment.org/2022/09/15/five-strategies-to-support-u.s.-democracy-pub-87918

[69] Wintemute GJ, Velasquez B, Shev AB, Tomsich EA, Wright MA, Reeping PM, et al. Fear, loathing, and support for political violence in the United States: findings from a nationally representative survey. Lancet Reg Health Am. 2025;51:101235. doi: 10.1016/j.lana.2025.101235 41019518 PMC12464958

[70] Wintemute GJ, Velasquez B, Li Y, Tomsich EA, Reeping PM, Robinson SL. Racist and pro-violence beliefs, approval of extreme right-wing political organizations and movements, and support for political violence in the United States. SocArXiv. 2023. doi: 10.31235/osf.io/c9vtr PMC12683887 41361497

