# Supplementary material for: Party affiliation, political ideology, views on democracy and society, and support for political violence in the United States of America: findings from a nationally representative survey
Source: PLoS One. 2026 Sep 16;21(9):e0355678. doi: 10.1371/journal.pone.0355678 (PMC13581029; doi:10.1371/journal.pone.0355678)
Supplement: Supplementary file 2 [file pone.0355678.s001.zip › Update to data contents file 20250328.docx]

Update to data contents file

20250328

Some tables were moved from the manuscript and renumbered during the peer review process. Table numbering in the data contents file was not changed. The filenames for individual data files were changed and reflect the final ordering. The table shows the changes.

| **Old numbering** | **New numbering** |
| --- | --- |
| Table 1 | Sup Table 5 |
| Table 2 | Table 1 |
| Table 3 | Sup Table 6 |
| Table 4 | Sup Table 7 |
| Table 5 | Sup Table 8 |
| Table 6 | Table 2 |
| Table 7 | Table 3 |
| Table 8 | Table 4 |
| Table 9 | Table 5 |
| Table 10 | Table 6 |
| Sup Table 1 | Sup Table 1 |
| Sup Table 2 | Sup Table 2 |
| Sup Table 3 | Sup Table 3 |
| Sup Table 4 | Sup Table 4 |
| Sup Table 5 | Sup Table 9 |
| Sup Table 6 | Sup Table 10 |
| Sup Table 7 | Sup Table 11 |
| Sup Table 8 | Sup Table 12 |
| Sup Table 9 | Sup Table 13 |
| Sup Table 10 | Sup Table 14 |
| Sup Table 11 | Sup Table 15 |
| Sup Table 12 | Sup Table 16 |
| Sup Table 13 | Sup Table 17 |
| Sup Table 14 | Sup Table 18 |
| Sup Table 15 | Sup Table 19 |
| Sup Table 16 | Sup Table 20 |
